# Development of a Fully Automated Method HS-SPME-GC-MS/MS for the Determination of Odor-Active Carbonyls in Wines: a “Green” Approach to Improve Robustness and Productivity in the Oenological Analytical Chemistry

**DOI:** 10.1021/acs.jafc.2c07083

**Published:** 2023-02-27

**Authors:** Maurizio Piergiovanni, Silvia Carlin, Cesare Lotti, Urska Vrhovsek, Fulvio Mattivi

**Affiliations:** † Center Agriculture Food Environment (C3A), University of Trento, San Michele all’Adige (TN) 38010, Italy; ‡ Center Research and Innovation, Edmund Mach Foundation, San Michele all’Adige (TN) 38010, Italy

**Keywords:** Volatile carbonyl compounds, HS-SPME, wine aging, accelerated aging, oxygen, oxidation, green analytical chemistry

## Abstract

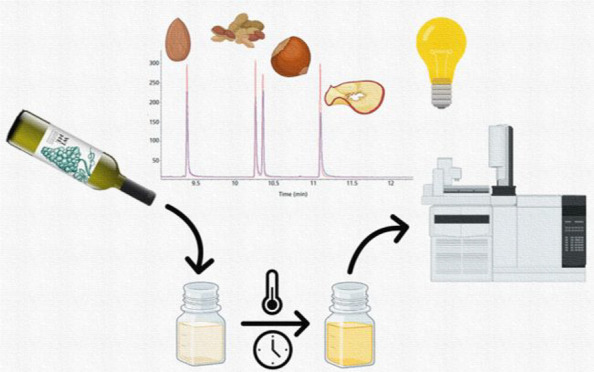

The aim of this study was the optimization and validation of a green, robust, and comprehensive method for the determination of volatile carbonyl compounds (VCCs) in wines that could be added as a new quality control tool for the evaluation of a complete fermentation, correct winemaking style, and proper bottling and storage. A HS-SPME-GC-MS/MS method was optimized and automated using the autosampler to improve overall performance. A solvent-less technique and a strong minimization of all volumes were implemented to comply with the green analytical chemistry principles. There were as many as 44 VCC (mainly linear aldehydes, Strecker aldehydes, unsaturated aldehydes, ketones, and many other) analytes under investigation. All compounds showed a good linearity, and the LOQs were abundantly under the relevant perception thresholds. Intraday, 5-day interday repeatability, and recovery performances in a spiked real sample were evaluated showing satisfactory results. The method was applied to determine the evolution of VCCs in white and red wines after accelerated aging for 5 weeks at 50 °C. Furans and linear and Strecker aldehydes were the compounds that showed the most important variation; many VCCs increased in both classes of samples, whereas some showed different behaviors between white and red cultivars. The obtained results are in strong accordance with the latest models on carbonyl evolution related to wine aging.

## Introduction

Oxygen plays a fundamental role in the production of fermented beverages because of its involvement in chemical reactions and biological processes that impact the sensory profile; among the products of these phenomena there are double bond carbon–oxygen compounds called carbonyls. These molecules are widely present in foods and beverages, as both aldehydes and ketones; their formation is due to chemical reactions such as Maillard reactions, Strecker degradation, aldol condensation, and lipid oxidation^1^ or biological processes like alcoholic fermentation.^2^ In some cases, they can also derive from raw materials or be released from wood barrels or toasted oak alternatives (chips, cubes, staves) during wine evolution and aging.^3^

Carbonyls, together with other volatile compounds, are responsible for the characteristic aromas of beer,^1^ spirits,^4^ wine,^5^ and, generally, for the production of all beverages where oxygen plays a key role.^6^ Due to their perception threshold comprised between tens of nanograms per liter to hundreds of micrograms per liter in most cases, carbonyls are perceptible despite their usually low concentrations.^7^ The presence of aromatic nuances of vanilla, caramel, butter, honey, potato, orange, lemon, violets, cider, and plum is the olfactory fingerprint of carbonyls.^8−15^ Since these are pleasant scents, the winemaking of Port,^16^ Sherry,^17^ Vin Santo,^18^ and Madeira^19^ is tailored to emphasize the production of these molecules.^20^ However, increased concentrations of some aldehydes with yeasty and oxidized scents are associated with wine oxidation. In most cases, oxidation and the related browning are long-standing problems that are commonly undesired and related to aroma defects.^21−23^

At bottling, oxygen, distributed in the headspace and dissolved into the wine, is usually present in negligible concentrations.^24^ However, that amount combined with the one that permeates through the closure, according to the oxygen transfer rate of the closure, and promoted by the exposure to fluctuation of temperature, can modify the oxidative status of the wine during its storage, with a consequent loss in varietal aroma and an increase in off flavors.^25^ Small amounts of oxygen at bottling can also promote the loss of sulfur dioxide via the sulfonation of several wine components.^26^ Both aldehydes and ketones are produced in the presence of oxygen, without any demonstrated difference in selectivity, even though aldehydes are the compounds most related to oxidative off-flavors.^25−28^

One of the most remarkable characteristics of this class of molecules is their reactivity, due to the presence of the carbonyl group; indeed, a high electrophilic carbon is suitable for nucleophilic additions such as the ones which take place with hydrogen sulfite (HSO_3_^–^), an equilibrium form of sulfur dioxide (SO_2_).^29,30^ The products of these reversible reactions are α-hydroxyalkylsulfonates, a class of nonvolatile compounds that does not contribute to wine aroma.^31^ Because of that, α-hydroxyalkylsulfonates behave like a “tank” that releases carbonyls during wine maturation and aging or under acidic conditions.^32^ As a result, the concentration of free sulfur dioxide at bottling can usually be enough to mask the presence of aldehydes, but their contribution can reappear during storage if the levels of free SO_2_ are depleted.

In red wines, molecules bearing electrophilic carbonyls, including acetaldehyde, can bind to flavonoids promoting the condensation of two flavonoids connected via an ethyl bridge and can react with the anthocyanins with the formation of wine pigments, such as the chemical class of vitisin B.^33^ Therefore, red wines, which are rich in polyphenols, are more resilient in the face of the development of oxidative flavors driven by aldehydes than white or rosé wines.

Based on the above, the concentration of carbonyls can be used for the evaluation of a complete fermentation and proper wine maturation and storage conditions.^34^ As a result, the quantitative determination of the volatile carbonyl content is very important, even though allowing the quantitation of these compounds at their subthreshold concentrations, requires the analytical method to be highly sensitive, selective, and robust. Most of the methods described in the literature involve a heterogeneous extraction, a derivatization, and are based on GC-MS techniques.^35^ Mayr et al. developed a GC-MS/MS quantitation method for 18 carbonyl compounds based on SPE extraction and O-(2,3,4,5,6-pentafluorobenzyl) hydroxylamine hydrochloride (PFBHA) derivatization on a cartridge.^36^ Even though this method shows excellent performance in terms of sensitivity and linearity, the SPE procedure is time-consuming and scarcely automatable, in contrast to the Green Analytical Chemistry rules.^37^ To overcome these limits, many other methods are based on the Head Space Solid Phase Micro Extraction technique (HS-SPME). This straightforward strategy does not involve any manual preliminary operation and combines high productivity and satisfactory performances.^38,42^ Many HS-SPME methods have been purposed with PFBHA on-fiber derivatization^39^ and in solution derivatization,^32,40^ both with satisfactory results but different ease of execution.^41^ Similar methods have also been used to perform carbonyl quantitation in other beverages, such as beer.^38^

To summarize, the amount of VCCs is a key parameter that could be used to monitor the state of the winemaking and, after the end of the vinification, the storing and bottling conditions to achieve the desired evolution. Because of that, the aim of this research was to optimize an analytical method that could be used as a quality control tool throughout the wine’s life. To do so, a fully automated HS-SPME method for the simultaneous quantification of 44 carbonyls with in-solution PFBHA derivatization was optimized; it was extensively validated in terms of linearity, intraday and interday repeatability, and recovery. The miniaturization of volumes and the use of a solvent-free technique, coupled with automation, have made it possible to obtain a method compliant with the green analytical chemistry principles, with a concurrent improvement in performance, repeatability, reliability, and productivity.

The obtained protocol is used to determine the effect of accelerated aging in several samples of red and white wines subjected to an accelerated aging procedure based on the one purposed by Pereira et al.;^43^ in this treatment, the samples are stored under controlled conditions and a relatively high temperature to unlock and speed up many of the transformations that occur during aging, making them effective in a few weeks, including oxidation and the formation of VCCs.

## Materials and Methods

### Solvents and Standards

All solvents for GC analysis (MS grade) were purchased from Merck KGaA (Darmstadt, Germany). Linear aldehydes (propanal, butanal, pentanal, hexanal, heptanal, octanal, nonanal, methional), E-2-unsaturated aldehydes (2-propenal, E-2-butenal, E-2-pentenal, E-2-hexenal, E-2-heptenal, E-2-octenal, E-2-nonenal, E-2-decenal), Strecker aldehydes (2-methylpropanal, 2-methylbutanal, 2-methylpentanal, 3-methyl-2-butenal, 3-methylbutanal, benzaldehyde, phenylacetaldehyde), ketones (2-butanone, 3-methyl-2-butanone, 2-pentanone, 3-pentanone, 3-penten-2-one, 2-hexanone, 3-hexanone, 2-methyl-3-pentanone, 2-cyclohexen-1-one, 2-heptanone, 4-heptanone, 2-octanone, 6-methyl-5-hepten-2-one, 2-nonanone, 2-decanone, 2-undecanone), and furans (2-furfural, 5-methyl-2-furfural) were purchased from Merck KGaA (Darmstadt, Germany). 3-Methylthio-2-butanone, 4-(methylthio)-2-butanone, 4-methyl-2-pentanone, and 4-methyl-4-methylthio-2-pentanone came from abcr GmbH (Karlsruhe, Germany). All standards were purchased at the highest purity available. A 1 g/L of ethanol solution of every compound was freshly prepared, and various mixtures of all analytes were prepared at lower concentration (10, 1, 0.1, and 0.01 mg/L), to allow every operation related to method optimization, calibration, and validation to be performed. A separate mixture of internal standards (acetone d6, 4-methyl-3-penten-2-one d10, octanal d16, and 4-fluorobenzaldehyde) was prepared in ethanol at 25 mg/L. The derivatizing solution was prepared at 40 g/L daily by dissolving solid PFBHA in water. SPME fibers (65 μm, bonded PDMS/DVB) came from Supelco/Merck KGaA (Darmstadt, Germany). Sodium metabisulfite and acetaldehyde were used to prepare SO_2_ and acetaldehyde solutions employed for derivatization studies and were purchased from Merck KGaA (Darmstadt, Germany).

### Samples

Several bottles of four fortified wines aged for 5 (Sherry and Madeira) and 10 years (Port and Marsala) were bought at the local wine shop and used to check the method performances. As many as 14 commercial wine samples from the Trentino — Alto Adige (Italy) regional production were selected for accelerated aging purposes. Several bottles of seven different wines from the white variety Gewürztraminer and seven from the red variety Teroldego were sampled. All samples were from the 2019 harvest and are reported in Table 1.

**Table 1:** Detailed List of Wines Submitted to the Accelerated Aging Procedure with Variety and Producer

sample	variety	geographical indication	producer
1G	Gewürztraminer	Alto Adige DOC	Elena Walch
2G	Gewürztraminer	Alto Adige DOC	Campaner
3G	Gewürztraminer	Alto Adige DOC	Abbazia Novacella
4G	Gewürztraminer	Alto Adige DOC	Flora
5G	Gewürztraminer	Alto Adige DOC	Kurtasch
6G	Gewürztraminer	Alto Adige DOC	Kleinstein
7G	Gewürztraminer	Alto Adige DOC	Sanct Valentin
1T	Teroldego	Vigneti delle Dolomiti IGT	Cantina Sociale di Avio
2T	Teroldego	Teroldego Rotaliano DOC	Cantina Marco Donati
3T	Teroldego	Teroldego Rotaliano DOC	Casata Monfort
4T	Teroldego	Teroldego Rotaliano DOC	Cavit
5T	Teroldego	Teroldego Rotaliano DOC	Cantina F.lli Zeni
6T	Teroldego	Teroldego Rotaliano DOC	Cantina Rotaliana
7T	Teroldego	Teroldego Rotaliano DOC	Fondazione Edmund Mach

A commercial white wine (Tavernello bianco) produced mainly using Trebbiano grapes was used for the calibration curve after treatment with Geosorb (Laffort, Bordeaux, France), 100 g/L, for volatile compound removal. A commercial red wine (Tavernello rosso) and white wine (Tavernello bianco) were used for validation purposes. A 2020 Müller Thurgau from Fondazione Edmund Mach (San Michele all’Adige (TN), Italy) and a 2017 Sfursat (a passito wine produced in Lombardia using Nebbiolo grapes) were used for the evaluation of recoveries in totally different matrices (young white and oxidized red wines).

### Accelerated Aging Procedure

All of the bottles were opened under an inert atmosphere inside a sealed hood provided by Captair Pyramid, fed with a continuous stream of nitrogen to ensure the absence of oxygen. Under those conditions, wines were split between a 2 mL amber vial for the analysis of the fresh sample and 2 × 100 mL glass bottles for the accelerated-aging process.

To determine the effect of gaseous oxygen and headspace, a preliminary couple of samples (one Gewürztraminer and one Teroldego) was subjected to the whole accelerated aging procedure (5 weeks and 50 °C) with different empty volumes (0, 5, 50, and 75 mL).

To determine the evolution of the VCCs during aging, seven Gewürztraminer and seven Teroldego samples were treated as follows: the bottles were filled to leave 0.7 mL of Head space to simulate real bottle conditions and stored at 50 °C, and samples were analyzed as they were (*t*_0_), after 2.5 weeks (*t*_m_), and after 5 weeks (*t*_f_), in randomized session. The oxygen amount was monitored daily using a NOMA Sense sensor (Wine Quality Solutions, Rodilhan, France). Finally, the treated samples were opened and the wine transferred into 2 mL amber vials stored at 4 °C.

### Instrumentation

All GC-MS analyses were carried out using a TSQ Quantum XLS Ultra Triple Quadrupole GC-MS/MS (Thermo Scientific, Austin, TX, Usa) equipped with a 30 m × 0.25 mm ID × 0.25 μm Restek Rx Sil MS w/Integra-Guard column (Restek corporation, Bellofonte, PA, USA). A split–splitless injector was set at 250 °C and programmed in splitless mode for the first 4 min after the injection to allow a complete desorption. The GC separation starts at 40 °C, is held for 4 min, then is increased with the following intervals: 40–80 °C at 20 °C/min, 4 min at 80 °C, 80–100 °C at 2 °C/min, 5 min at 100 °C, 100–170 °C at 2.5 °C/min, and finally 170–250 at 20 °C/min with a 1 min final isotherm at 250 °C. A 1.2 mL/min helium was the carrier gas of choice. The MS signal was obtained by electron ionization at 70 eV, with the transfer line and the ion source both set at 250 °C; MRM acquisition mode was used to ensure the best sensibility and specificity. A CTC-PAL3 autosampler was used for performing preparation, extraction, and injection. The instrument was operated with XCalibur software, the sample preparation sequence was handled using TriPlus RSH Sampling Workflow Editor, and all of the analytical data were processed using Tracefinder, all provided by Thermo Scientific (Thermo Scientific, Austin, TX, USA).

### Sample Preparation

**Figure 1:**
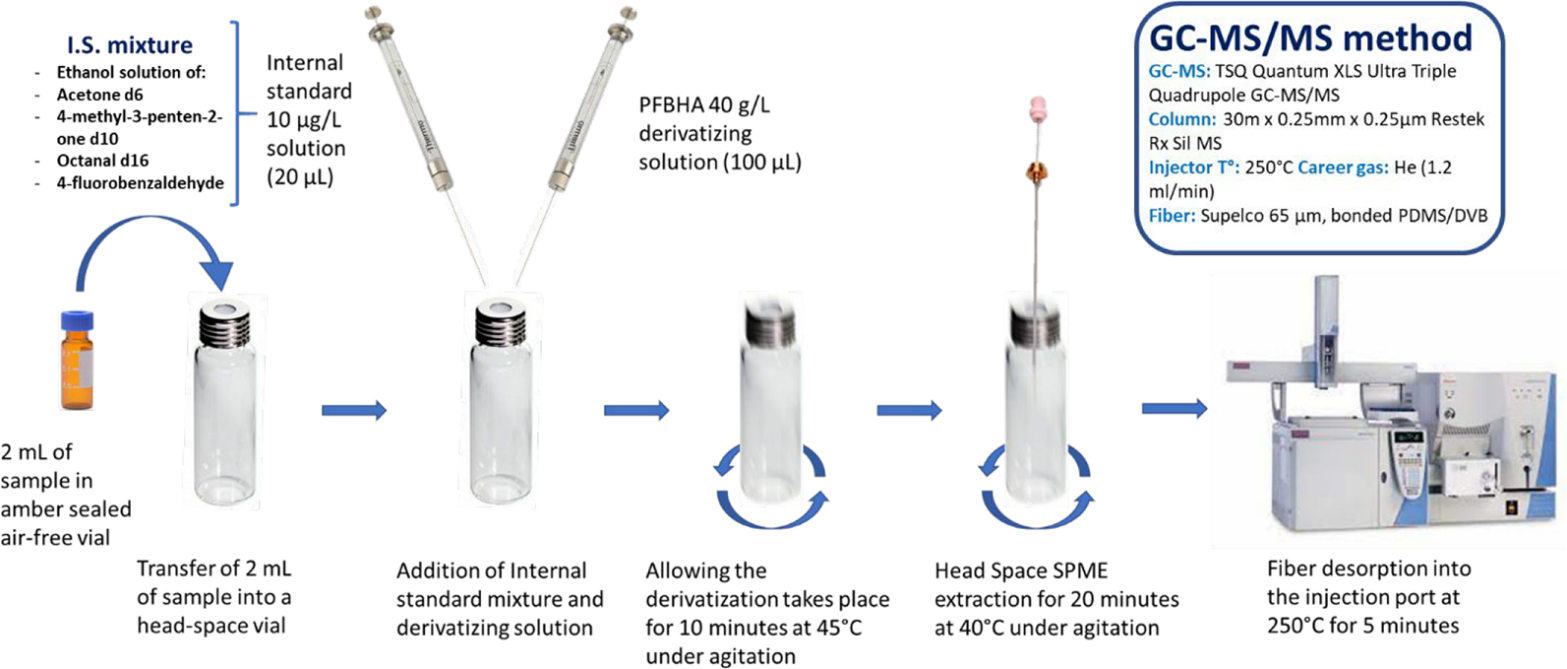
Step-by-step workflow of the automated sample preparation.

The extraction procedure was based on the one purposed by Moreira et al.,^44^ upgraded with a fully automated sample preparation. All operations were entirely carried out by the autosampler where all loaded vials were kept in a 5 °C cooled tray holder and are shown in Figure 1. The sample was first transferred from the 2 mL vial to a 20 mL head space vial, and then spiked with 20 μL of 10 μg/L internal standard solution and 100 μL of 40 g/L PFBHA solution. Next, the vial was moved into a 45 °C heated stirrer (300 rpm) where the derivatization reaction takes place. In the meanwhile, the SPME fiber was conditioned at 270 °C for 5 min in the conditioning station. When the derivatization process was finished, the vial was moved into a second 40 °C heated stirrer (250 rpm) where it was conditioned for 5 min and extracted with the SPME for 20 min. Finally, the SPME fiber was moved into the injector and exposed at 250 °C for 4 min. The autosampler was programmed to work while the chromatography was running to make the whole procedure as efficient as possible and prevent sample degradation.

### GC-QqQ-MS Analysis Conditions

Retention times and MRM transitions are reported in Table 2. Parent ions and product ions were determined manually by various GC-MS/MS experiments on pure analytes; in the end, collision energies were optimized. Due to the matrix complexity, parent and product ions were selected among those which have fewer matrix interferents and lower background noise, rather than due to their intensity.

**Table 2:** Acquisition Program with Retention Times, Calibration Range, and MRM Quantifier and Qualifier Transitions for All Analytes and Internal Standards^a^

analyte	Rt (min)	quantifier (*Q*)	qualifier (*q*)	calibration curve (μg/L)
acetone d6 (I.S.)	17.33	259→181(15)	259→212(5)	
2-propenal	19.08	250→181(10)	181→161(5)	0.059–1175
propanal	18.28	239→181(15)	239→207(5)	0.050–1007
2-methylpropanal	20.54	267→181(10)	250→181(10)	0.056–1125
2-butanone	21.8	250→181(15)	181→161(5)	0.046–925
butanal	24.91	239→181(15)	239→207(5)	0.050–1000
3-methyl-2-butanone	25.58	253→181(15)	253→177(5)	0.050–1000
3-pentanone	27.2	181→161(5)	264→181(15)	0.050–996
2-pentanone	27.21	253→181(15)	253→177(5)	0.050–1005
2-methylbutanal	28.09	239→181(15)	239→207(5)	0.050–1009
3-methylbutanal	28.73	239→181(15)	239→207(5)	0.040–800
E-2-butenal	28.95	250→181(10)	250→250(5)	0.050–99.4
2-methyl-3-pentanone	29.49	295→114(5)	72→54(10)	0.050–1003
4-methyl-2-pentanone	30.11	253→181(15)	253→177(5)	0.055–1100
pentanal	31.32	239→181(15)	239→207(5)	0.051–1011
3-hexanone	31.95	250→181(10)	181→161(5)	0.050–1005
2-methylpentanal	32.41	253→181(15)	239→181(15)	0.050–990
2-hexanone	32.99	253→177(5)	253→181(15)	0.058–1150
3-penten-2-one	33.26	264→181(15)	181→161(5)	0.050–993
4-methyl-3-penten-2-one d10 (I.S.)	33.3	285→181(10)	285→285(5)	
E-2-pentenal	35.48	250→181(10)	181→161(5)	0.050–100
3-methyl-2-butenal	36.44	264→181(15)	264→161(15)	0.050–1001
hexanal	37.16	181→161(15)	239→181(15)	0.050–997
2-heptanone	38.24	253→181(15)	253→177(5)	0.050–1000
2-furfural	38.58	291→181(10)	291→249(5)	0.051–5070
3-methylthio-2-butanone	39.98	181→161(15)	267→86(10)	0.051–1010
E-2-hexenal	40.93	250→181(10)	181→161(5)	0.050–99.7
heptanal	42.15	181→161(15)	239→181(15)	0.050–994
6-methyl-5-hepten-2-one	42.65	82→67(15)	82→82(5)	0.049–988
2-octanone	42.95	181→161(15)	253→181(15)	0.051–1011
2-cyclohexen-1-one	42.96	274→181(15)	274→274(5)	0.050–998
methional	43.58	252→181(10)	252→252(5)	0.049–983
4-(methylthio)-2-butanone	44.11	266→181(15)	266→266(5)	0.061–1225
5-methyl-2-furfural	44.32	181→161(5)	305→181(15)	0.051–5085
E-2-heptenal	45.49	250→181(10)	181→161(5)	0.050–100
octanal d16 (I.S.)	46.77	181→161(15)	243→181(15)	
octanal	46.79	181→161(15)	239→181(15)	0.050–1007
4-fluorobenzaldehyde (I.S.)	47.19	181→161(10)	319→181(5)	
2-nonanone	47.4	181→161(15)	253→181(15)	0.050–996
benzaldehyde	47.46	301→181(15)	301→271(5)	0.066–1325
4-methyl-4-methylthio-2-pentanone	48.43	181→161(15)	294→181(15)	0.055–1100
phenylacetaldehyde	49.76	181→161(5)	91→65(15)	0.050–1002
E-2-octenal	49.89	250→181(10)	181→161(5)	0.050–101
nonanal	51.07	181→161(15)	239→181(15)	0.050–990
2-decanone	51.6	181→161(15)	253→181(15)	0.055–1100
E-2-nonenal	53.72	250→181(10)	181→161(5)	0.059–118
2-undecanone	54.6	253→181(15)	253→177(5)	0.050–997
E-2-decenal	55.47	250→181(15)	181→161(5)	0.060–120

a Transitions are expressed as parent ion → product ion (collision energy, eV).

### Calibration Curve Acquisition

Calibration curves were acquired from 0.05 μg/L to 1000 μg/L, except for furans, where it was extended up to 5000 μg/L. Calibration samples were prepared spiking the treated commercial wine (Tavernello) mentioned in the “Samples” section, with the analyte mixtures prepared as indicated in the “Solvents and Standards” section. All curves were interpolated from 0.05 to 250 μg/L, which is the concentration range where most analytes belong; higher points were included only if needed. The calibration range is reported in Table 2; the first value of the curve (LOQ) has, for all analytes, a signal-to-noise ratio (S/N) > 10 calculated for the qualifier (*q*) transition response. *R*^2^ > 0.99 was the acceptance criterion for allowing a curve to be used in the quantitation method.

### Optimization of the Derivatization Step

To evaluate the optimal conditions for derivatization, two of the white wines used for accelerated aging were chosen from among those with the highest and lowest sulfur dioxide (1G and 2G, respectively). These samples were subsequently divided into aliquots which were in turn analyzed at different derivatization times (5, 10, 15, 20, 30, 60, and 120 min) without modifications, added with SO_2_ (spike of supplementary 20 mg/L), and supplemented with acetaldehyde (spike of supplementary 40 mg/L). Spikes of SO_2_ and acetaldehyde were made 2 days before analysis to allow the wine sample to reach its steady state, whereas all measurements were acquired within the same batch in randomized sessions. This study was performed analyzing new bottles from the same batch but opened several months later so differences in concentrations compared to results reported for the aging experiments should be addressed to this fact. All samples were acquired in duplicate.

### Method Validation Procedure

The procedure was validated in terms of repeatability and recovery; repeatability was evaluated at 0.2, 5, and 50 μg/L analyzing laboratory samples (same matrix used for the calibration curve spiked with a reprepared analyte solution) intraday (five replicates within a day) and interday (six replicates in different days within a week) and a commercial red wine spiked with the same procedure. Intraday repeatability was also evaluated for a commercial sample by analyzing an untreated 2020 Müller-Thurgau. Recovery was evaluated spiking the Müller-Thurgau at 5 μg/L with the same analyte solution used for the calibration curve and analyzing it in triplicate. Calculations were made with the following formula:
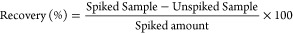


### Sample Analysis

All samples were loaded at the same time and analyzed in randomized order; randomization was done using Microsoft Excel. Metrological traceability was assessed by analyzing a Continuous Calibration Verification (CCV) at 5 μg/L each 10 samples after a fiber blank (FB). FB is a sample acquired with an empty vial without adding derivatizing solution and internal standards just for a periodic fiber extra cleaning. CCV was a laboratory sample prepared with the same matrix used for the calibration curve but spiked with an independently prepared analyte solution. Acceptance criteria were ±20% for the internal standards area and ±30% for the analyte concentration; if one of these criteria was not satisfied, the following CCV was prepared from a fresh solution. If it was unsatisfactorily the same, ±30% analytes were accepted as “semi-quantitative” and ±50% were rejected and analyzed again with a new calibration curve. Sequences were made of 10 sample sections as follows:

- FB
- CCV
- Sample 1
- ...
- Sample 10
- FB
- CCV

### Statistical Analysis

Statistical analysis was done using Metaboanalyst^45^ (https://www.metaboanalyst.ca/home) for ANOVA and CAT^46^ (Chemometric Agile Tool, http://gruppochemiometria.it/index.php/software) software for chemometrics.

## Results and Discussion

### Method Performance and Validation

The method for the determination of VCCs used in this research was mostly based on the one developed by Moreira et al. adapted and optimized for use with GC-MS/MS instead of GC-IT/MS (ion trap mass spectrometry).^44^ In addition, a supplementary evaluation was performed to better study derivatization times which can be crucial because of the presence of SO_2_ in wine samples. In this experiment, samples were analyzed after different derivatization times without any treatment, with a 20 mg/L addition of SO_2_ and with a 40 mg/L addition of acetaldehyde. Results for those compounds detectable in both samples are reported in Figures S1 and S2. Since the calibration curve was acquired with a derivatization time of 10 min, results were expressed as a ratio of compound area vs internal standard area instead of micrograms per liter. Because of the relative youngness of these samples, only 10 VCCs were detectable in both samples working with a derivatization time of 10 min. Results showed two different major trends, depending on the characteristics on the analytes. Smaller molecules (2-butanone, 2-methylpropanal, 2-pentanone, 3-hexanone, 3-pentanone, propanal, and butanal) gave a maximum response close to 10 min and then decreased for times longer than 30 min. On the other hand, bigger and heavier compounds (2-furfural, 2-nonanone, and 5-methyl-2-furfural) showed a lower time dependency and a smoothed maximum of efficiency. In both samples and for all detected analytes, a 10 min derivatization time was the best compromise to obtain a good response.

The spikes of SO_2_ or acetaldehyde did not affect trends observed, and the differences were minimal and consistent with uncertainty. In fact, SO_2_ addition aimed to evaluate the reversibility of the VCC-sulfur dioxide interaction since its increase at massive concentrations bonded free carbonyls, minimizing their concentration. Since area ratios with and without the addition were very similar, it was demonstrated that the derivatization process was able to strongly shift the equilibrium toward the derivatized form and α-hydroxyalkylsulfonates were hydrolyzed to release carbonyls for the reaction with the PFBHA. A recent study by Ferreira et al., published after all of the experimental activity reported in this paper, evaluated the effects due to SO_2_ in the derivatization of five Strecker aldehydes^47^ (Castejón-Musulén et al.). In this research, the authors studied the reaction evolution using two different PFBHA concentrations (0.21 and 0.3 g/L) with and without an SO_2_ addition. Interestingly, the authors noticed some differences due to sulfur dioxide in the shorter time steps (2 and 5 h), whereas those gaps decreased strongly in longer periods (12 and 24 h). This result supported the use of PFBHA at higher concentrations, which was able to shift the derivatization reaction faster toward the derivatized form. In fact, assuming the reaction between PFBHA and the carbonyl function is a first order kinetic as demonstrated by Pawliszyn et al. for formaldehyde,^48^ using a 40 g/L PFBHA concentration (from 190 to 133 times higher than 0.21 or 0.3 g/L) could be a valuable modification to make the process give a satisfactory efficiency after 10–15 min.

On the contrary, acetaldehyde is the smallest VCC, and its addition in high concentration was used to make this molecule subtract SO_2_ present in wine and release bound VCCs, increasing their concentration in free form. In addition, because of this reaction, it should be expected that some of the 34 VCCs below the LOQ became free and detectable. Experimental results were not affected by this treatment as well as for the previous one, so it was demonstrated that the reaction with PFBHA at 40 g/L was strongly shifted toward the derivatized forms and was able to react with both free and bound carbonyls. In conclusion, 10 min was the derivatization time, which allowed measurement of the total concentration of VCCs with also a good compromise in terms of response.

About validation, the method was first evaluated in terms of linearity, which was satisfactory (*R*^2^ > 0.99) for all analytes from approximately 0.05 μg/L to 250 μg/L; since most VCCs are usually comprised in this range, calibration points over 250 μg/L were interpolated only if the sample has a measured amount over this value. LOQs were identified as the first calibration level (0.05 μg/L), whereas LODs were not calculated, since the perception threshold of all analytes^49−52^ was considerably higher than the analytical detection limit.

Repeatability data show satisfactory results for all analytes, both intraday and interday at all concentration levels. For the intraday analyses, most compounds at 5 μg/L were comprised between 3.5 μg/L and 6 μg/L, most with an RSD lower than 20% and only for two analytes higher than 30%, demonstrating good precision (Table 3). A very similar trend was revealed at the higher level, since only a few analytes have measured concentrations out of 50 ± 30% μg/L (most on the lower side), mainly due to the proximity to the end of the linearity range for those compounds (Table 4). At 50 μg/L, RSDs are noticeably lower, and precision was considerably better.

**Table 3:** Intraday and Interday Repeatability for Laboratory Sample Prepared at 5 μg/L

	intraday repeatability		interday repeatability	
analyte	avg. conc.	RSD (%)	avg. conc.	RSD (%)
2-butanone	4.40	5.32	4.66	29.58
2-cyclohexen-1-one	6.36	32.70	5.71	27.89
2-decanone	7.95	28.03	9.14	18.86
2-furfural	3.81	32.16	4.61	33.16
2-heptanone	4.09	19.37	3.89	20.82
2-hexanone	5.35	9.62	6.01	14.65
2-methyl-3-pentanone	5.09	1.38	5.13	17.04
2-methylbutanal	4.36	2.79	5.16	20.47
2-methylpentanal	4.21	4.70	4.53	15.67
2-methylpropanal	3.64	4.82	3.83	30.82
2-nonanone	5.09	23.85	5.47	13.73
2-octanone	4.29	16.54	4.48	11.94
2-pentanone	4.97	5.12	5.45	31.53
2-propenal	3.56	16.10	3.90	30.56
2-undecanone	6.07	25.27	7.85	33.36
3-hexanone	4.95	4.39	5.21	15.84
3-methyl-2-butanone	4.01	2.48	4.26	15.62
3-methyl-2-butenal	3.51	18.00	3.27	19.48
3-methylbutanal	3.76	5.85	7.04	31.01
3-methylthio-2-butanone	4.15	16.22	4.30	8.02
3-pentanone	4.91	3.27	4.98	17.28
4-(methylthio)-2-butanone	4.13	13.66	4.48	6.62
4-heptanone	5.74	10.31	5.33	16.88
4-methyl-2-pentanone	4.46	3.71	4.64	16.21
4-methyl-4-methylthio-2-pentanone	5.36	13.48	5.49	4.79
5-methyl-2-furfural	5.73	19.25	8.07	28.57
6-methyl-5-hepten-2-one	4.29	21.69	4.93	12.40
benzaldehyde	4.10	29.74	5.48	17.41
butanal	4.64	12.50	3.69	26.80
E-2-butenal	3.72	12.96	4.01	31.08
E-2-decenal	3.74	23.31	3.70	30.05
E-2-heptenal	3.93	18.23	3.45	27.80
E-2-hexenal	3.56	13.60	3.22	22.52
E-2-nonenal	4.03	20.30	3.67	27.00
E-2-octenal	4.38	19.03	3.92	26.25
E-2-pentenal	3.87	16.11	3.32	25.82
heptanal	3.71	12.91	3.88	30.21
hexanal	3.53	29.27	3.99	29.24
methional	4.47	29.72	6.27	26.33
nonanal	3.29	8.67	3.45	33.80
octanal	4.20	22.25	3.86	32.40
pentanal	3.99	17.98	3.71	29.23
phenylacetaldehyde	4.53	17.01	4.05	22.52
propanal	5.40	5.52	6.41	32.24

**Table 4:** Intraday and Interday Repeatability for Laboratory Sample Prepared at 50 μg/L

	intraday repeatability		interday repeatability	
analyte	avg. conc.	RSD (%)	avg. conc.	RSD (%)
2-butanone	51.58	9.65	49.09	26.21
2-cyclohexen-1-one	57.92	16.07	49.20	9.26
2-decanone	63.68	4.30	63.17	20.96
2-furfural	48.56	24.75	51.08	31.34
2-heptanone	51.88	12.48	45.14	21.26
2-hexanone	66.61	11.18	63.86	29.47
2-methyl-3-pentanone	66.36	11.95	58.40	28.11
2-methylbutanal	56.24	10.49	56.02	20.97
2-methylpentanal	55.87	11.14	54.30	29.97
2-methylpropanal	64.01	10.06	60.05	29.87
2-nonanone	36.66	9.86	36.15	15.35
2-octanone	40.96	15.43	36.66	19.85
2-pentanone	58.92	10.91	53.95	25.19
2-propenal	34.09	16.72	22.17	25.27
2-undecanone	43.42	6.00	43.23	24.85
3-hexanone	64.54	12.01	56.76	28.38
3-mercapto-2-pentanone	65.05	24.34	85.19	28.02
3-methyl-2-butanone	58.92	10.53	53.96	28.41
3-methyl-2-butenal	44.38	11.72	31.04	30.17
3-methylbutanal	45.91	16.47	47.55	25.87
3-methylthio-2-butanone	50.11	13.67	42.61	13.30
3-pentanone	60.23	10.39	52.37	29.97
4-(methylthio)-2-butanone	68.38	19.00	62.39	8.66
4-methyl-2-pentanone	60.56	11.98	54.44	29.26
4-methyl-4-methylthio-2-pentanone	62.11	12.52	60.72	6.75
5-methyl-2-furfural	52.56	21.00	55.16	18.41
6-methyl-5-hepten-2-one	57.16	14.98	51.77	16.33
benzaldehyde	42.79	11.38	46.28	7.34
butanal	49.02	7.29	49.06	35.27
E-2-butenal	56.19	11.85	40.59	26.05
E-2-decenal	41.20	11.75	35.64	28.31
E-2-heptenal	48.67	21.53	33.25	29.01
E-2-hexenal	36.74	11.65	27.43	28.34
E-2-nonenal	31.37	6.62	23.70	26.35
E-2-octenal	32.80	4.63	24.78	27.32
E-2-pentenal	45.77	9.58	32.68	28.78
heptanal	33.09	10.64	26.07	20.25
hexanal	44.76	24.91	27.92	29.63
methional	52.01	27.80	59.19	30.62
nonanal	46.76	11.67	38.45	30.30
octanal	49.17	1.54	40.25	14.48
pentanal	48.96	11.00	38.86	25.03
phenylacetaldehyde	32.24	5.55	24.41	27.43
propanal	63.30	8.85	64.25	28.01

Similar assumptions can be made for interday results at both concentrations, but in this case, most RSDs were comprised between 20% and 30%. These results demonstrate a very good reliability, especially considering that all samples are not prepared in model wine but in a real matrix, so the changes made to maintain balance can modify concentrations during the week.

Intra- and interday repeatability were evaluated also at 0.2 μg/L, and the results are reported in Table S1. As well as for 5 and 50 μg/L, at the lowest concentration level (far under the perception threshold), repeatability was satisfactory. In this case, average concentration values were in some cases different from the spiked concentration because of the use of a wine instead of model wine as a matrix. Despite the careful storage conditions, variation of some concentration which can be intended as negligible at 5 μg/L and 50 μg/L emerged at 0.2 μg/L. However, this predictable behavior did not affect repeatability, which was satisfactory.

The same validation assay was repeated in a red wine, which was a commercial product produced mainly from Sangiovese grapes. Like for the white one, intra- and interday repeatability were evaluated at 0.2, 5, and 50 μg/L, and results are reported in Table S2. Variations were like the ones detected for the white wine and, in some cases, even better, thanks to the higher stability due to the red matrix.

Comparable results are recorded for the intraday repeatability of the Müller–Thurgau (Table 5) and Sfursat samples (Table S3). Internal standard areas (data not shown) have RSDs comprised between 4.86% (4-methyl-3-penten-2-one d10) and 17.58% (4-fluorobenzaldehyde), which confirms their satisfactory stability.

**Table 5:** Intraday Repeatability for a Real Wine and Recovery Evaluation of the Same Sample Spiked at 5 μg/L

	Müller-Thurgau		Müller-Thurgau + spike 5 μg/L		
analyte	avg. conc.	RSD (%)	avg. conc.	RSD (%)	recovery %
2-butanone	3.95	20.09	9.52	7.20	111.36
2-cyclohexen-1-one	<0.05	n.a.	5.15	2.62	102.91
2-decanone	<0.05	n.a.	4.60	25.22	98.48
2-furfural	40.24	0.74	47.27	11.39	140.59
2-heptanone	<0.05	n.a.	3.17	6.55	112.20
2-hexanone	0.31	0.32	4.58	4.12	85.43
2-methyl-3-pentanone	0.76	0.00	4.36	4.99	71.89
2-methylbutanal	<0.05	n.a.	5.68	8.92	114.14
2-methylpentanal	<0.05	n.a.	3.83	1.81	80.78
2-methylpropanal	1.79	18.40	7.87	6.16	121.64
2-nonanone	<0.05	n.a.	4.68	6.63	120.27
2-octanone	<0.05	n.a.	4.79	2.34	107.60
2-pentanone	11.27	6.05	16.84	3.81	111.33
2-propenal	<0.05	n.a.	1.82	24.50	111.15
2-undecanone	<0.05	n.a.	5.03	25.61	140.26
3-hexanone	0.15	14.57	3.83	2.85	73.56
3-methyl-2-butanone	0.15	8.53	4.27	4.61	82.51
3-methyl-2-butenal	<0.05	n.a.	3.97	1.73	88.73
3-methylbutanal	4.25	22.12	9.28	4.65	100.63
3-methylthio-2-butanone	0.65	0.00	4.96	3.98	86.09
3-pentanone	5.18	5.35	8.96	21.96	75.64
3-penten-2-one	<0.05	n.a.	6.09	5.71	129.53
4-(methylthio)-2-butanone	0.00	n.a.	4.30	7.76	86.01
4-methyl-2-pentanone	0.38	1.33	4.30	4.32	78.39
4-methyl-4-methylthio-2-pentanone	6.16	8.71	11.51	12.05	107.07
5-methyl-2-furfural	<0.05	n.a.	2.76	5.84	125.99
6-methyl-5-hepten-2-one	<0.05	n.a.	4.35	1.28	107.89
benzaldehyde	3.48	10.85	10.28	5.19	136.01
butanal	0.16	29.37	6.12	16.96	119.21
E-2-butenal	<0.05	n.a.	2.61	4.47	100.77
E-2-decenal	<0.05	n.a.	4.50	7.14	146.77
E-2-heptenal	<0.05	n.a.	4.38	8.06	95.26
E-2-hexenal	<0.05	n.a.	4.29	1.81	89.03
E-2-nonenal	<0.05	n.a.	4.61	21.24	104.82
E-2-octenal	<0.05	n.a.	5.13	16.54	114.19
E-2-pentenal	<0.05	n.a.	4.01	3.82	81.43
heptanal	<0.05	n.a.	2.76	7.21	81.29
hexanal	<0.05	n.a.	5.56	21.65	120.75
methional	<0.05	n.a.	3.93	15.79	102.79
nonanal	2.05	7.19	8.04	20.46	119.80
octanal	<0.05	n.a.	3.78	8.42	75.52
pentanal	<0.05	n.a.	3.49	17.38	106.18
phenylacetaldehyde	<0.05	n.a.	5.43	12.87	125.62
propanal	2.78	0.56	9.22	10.72	128.81

A very similar trend was registered for recoveries, since all analytes except 2-furfural, 2-undecanone, benzaldehyde, and E-2-decenal had a recovery of 100 ± 30% as reported in Table 5. These results confirm the stability and reliability of the method and, in addition, demonstrate that, thanks to the choice of internal standards and the optimization of chromatography, the matrix effect is negligible. In the Sfursat sample, which was a completely different matrix, the same study was performed at both 5 and 50 μg/L. Recoveries were comprised in the same range described above (100 ± 30%) even though most analytes were within ±20%.

The method was finally tested in the analysis of four fortified wines, which were expected to be rich in VCCs and could be assumed as a tricky matrix; results are reported in Table 6. Such results are in good accordance with literature data^17,28,40,53^ and confirm the method’s suitability also for carbonyl-rich samples. Extraction conditions and calibration were tailored for the analysis of wines (young and aged), so it was expected that some analytes’ concentrations would be above the highest calibration point for these samples.

**Table 6:** Concentration of VCCs in Fortified Wine Samples in μg/L^a^

analyte\sample name	Sherry (5 years)	Madeira (5 years)	Marsala (10 years)	Port (10 years)
2-butanone	1190* ± 57	715 ± 34	200 ± 11	812 ± 39
2-cyclohexen-1-one	0.25 ± 0.02	0.3 ± 0.02	0.36 ± 0.03	0.31 ± 0.04
2-decanone	<0.05	<0.05	0.13 ± 0.04	<0.05
2-furfural	5186* ± 642	9892* ± 1224	5128* ± 635	5707* ± 706
2-heptanone	<0.05	1.97 ± 0.12	2.97 ± 0.19	2.3 ± 0.14
2-hexanone	0.13 ± 0.03	0.36 ± 0.04	0.29 ± 0.05	0.25 ± 0.05
2-methyl-3-pentanone	2.25 ± 0.13	0.21 ± 0.05	<0.05	1.95 ± 0.12
2-methylbutanal	8940* ± 469	1503* ± 79	467 ± 25	1501* ± 78
2-methylpentanal	4.07 ± 0.23	1.22 ± 0.07	0.42 ± 0.05	1.01 ± 0.06
2-methylpropanal	1090* ± 58	629 ± 32	175 ± 9	738 ± 37
2-nonanone	0.91 ± 0.04	0.63 ± 0.05	3.39 ± 0.17	2.72 ± 0.13
2-octanone	<0.05	0.17 ± 0.01	0.23 ± 0.02	0.28 ± 0.02
2-pentanone	128 ± 7	35.8 ± 1.9	27.1 ± 1.4	60.1 ± 3.8
2-propenal	15932* ± 1332	2591* ± 217	1333* ± 111	2460* ± 206
2-undecanone	0.25 ± 0.03	0.12 ± 0.04	0.63 ± 0.04	<0.05
3-hexanone	3.49 ± 0.21	1.77 ± 0.12	1.56 ± 0.09	6.59 ± 0.4
3-methyl-2-butanone	27.6 ± 3.3	55.4 ± 6.74	5.77 ± 0.29	58.5 ± 7.1
3-methyl-2-butenal	1.18 ± 0.16	6.11 ± 0.32	0.54 ± 0.09	1.8 ± 0.29
3-methylbutanal	71973* ± 4218	21838* ± 1280	5188* ± 304	11974* ± 702
3-methylthio-2-butanone	<0.05	<0.05	<0.05	0.14 ± 0.01
3-pentanone	35.7 ± 2.4	17.1 ± 1.1	18.8 ± 1.8	25.8 ± 1.7
4-(methylthio)-2-butanone	0.22 ± 0.04	<0.05	<0.05	<0.05
4-heptanone	0.15 ± 0.04	0.25 ± 0.02	<0.05	<0.05
4-methyl-2-pentanone	3.62 ± 0.22	4.25 ± 0.25	0.8 ± 0.05	5.8 ± 0.35
4-methyl-4-methylthio-2-pentanone	3.46 ± 0.22	0.66 ± 0.08	2.77 ± 0.17	0.81 ± 0.15
5-methyl-2-furfural	611 ± 64	144 ± 15	334* ± 35	87.7 ± 9.2
6-methyl-5-hepten-2-one	<0.05	<0.05	<0.05	<0.05
benzaldehyde	53.8 ± 3.6	493 ± 28	156 ± 8	261 ± 19
butanal	1228* ± 47	256 ± 9	51.6 ±1.9	157 ± 6
E-2-butenal	971 ± 58	900 ± 53	61.2 ± 3.6	202 ± 12
E-2-decenal	<0.05	<0.05	8.91 ± 0.62	0.61 ± 0.04
E-2-heptenal	0.24 ± 0.07	<0.05	1.23 ± 0.13	0.39 ± 0.14
E-2-hexenal	0.12 ± 0.04	0.15 ± 0.01	0.13 ± 0.04	<0.05
E-2-nonenal	<0.05	<0.05	2.06 ± 0.17	<0.05
E-2-octenal	<0.05	<0.05	1.55 ± 0.34	0.12 ± 0.04
E-2-pentenal	<0.05	<0.05	<0.05	<0.05
heptanal	5.59 ± 0.63	4.02 ± 0.51	10.8 ± 0.7	3.29 ± 0.37
hexanal	115 ± 14	138 ± 17	237 ± 25	63.4 ± 7.8
methional	42.3 ± 5.9	5.78 ± 1.8	69.5 ± 9.7	0.99 ± 0.24
nonanal	9.32 ± 0.54	2.13 ± 0.12	157 ± 9	13.9 ± 0.8
octanal	1.54 ± 0.21	0.92 ± 0.11	21.4 ±0.2	1.97 ± 0.02
pentanal	1028* ± 56	280 ± 15	90.4 ± 4.9	177 ± 9
phenylacetaldehyde	18.3 ± 2.5	24.7 ± 4.6	28 ± 5.8	6.27 ± 1.7
propanal	16378* ± 725	2563* ± 113	841 ± 37	2519* ± 111

a Values with * were semi-quantified over the maximum point of the calibration curve. All samples were analyzed in triplicate.

### Accelerated Aging Results

To understand the role of headspace volume, a dedicated preliminary accelerated aging experiment was performed by storing one sample from Gewürztraminer (G) and one from Teroldego (T) cultivars at 50 °C in 100 mL glass bottles fully filled (00), with 5 mL (05), 50 mL (50), and 75 mL (75) of free space; after 5 weeks samples were analyzed and the results for analytes whose variation was significant are reported in Table S4.

Interestingly, 15 VCCs, including 2-decanone, 2-methyl-3-pentanone, 2-nonanone, 2-undecanone, 3-methylthio-2-butanone, 4-heptanone, 6-methyl-5-hepten-2-one, E-2-decenal, E-2-heptenal, E-2-nonenal, E-2-octenal, E-2-pentenal, nonanal, and octanal did not change their concentration either in Gewürztraminer or in Teroldego wines, suggesting that, for these cultivars, the formation pathway of these carbonyls does not involve molecular oxygen and is not related to aging. As for the remaining VCCs, some of them showed a very strong increase for both cultivars (2-butanone (ethyl methyl ketone), 2-methylbutanal (2-methylbutyraldehyde), 2-methylpropanal (isobutyraldehyde), 2-pentanone (methyl propyl ketone), 2-propenal (acrolein), 3-methyl-2-butanone (methyl isopropyl ketone), 3-methylbutanal (isovaleraldehyde), butanal, hexanal, phenylacetaldehyde and propanal) confirming their role of benchmark oxidation products. Other compounds, such as 2-furfural, 2-methylpentanal, 4-methyl-2-pentanone, benzaldehyde, E-2-butenal, heptanal, methional, and pentanal, were the analytes whose formation was characterized by a binary behavior, since their increase in concentration is relevant only with 50 and 75 mL of air volume. Within the analyte set considered, the carbonyls which accumulated most were linear aldehydes, Strecker aldehydes, and furans, from three to seven carbons, in accordance with data in the literature.^32,54,55^ Finally, among all of the analytes quantified there were some which were not mentioned previously (2-cyclohexen-1-one, 2-heptanone, 2-hexanone, 2-octanone, 3-hexanone, 3-methyl-2-butenal, 3-pentanone, 4-(methylthio)-2-butanone, 4-methyl-4-methylthio-2-pentanone, 5-methyl-2-furfural, E-2-hexenal). The concentration of these carbonyls increased with head space volume, but slightly less than others, suggesting a formation pathway related to oxygen associated with a slower kinetic or a low abundance of related precursors.

**Figure 2:**
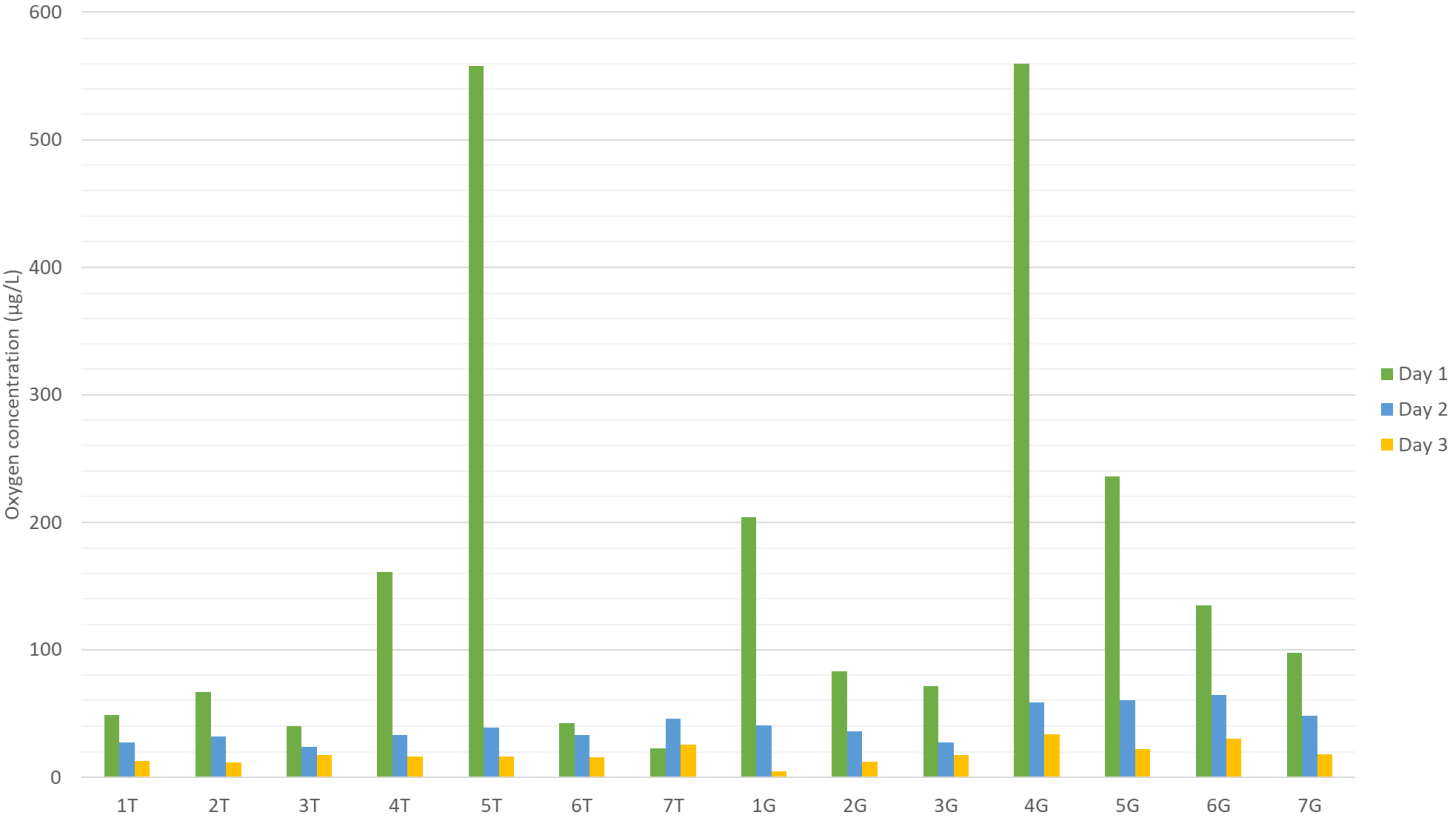
Measured oxygen amount in wine samples during the first 3 days of accelerated aging.

Concerning the accelerated aging experiments, both white and red samples were monitored in terms of molecular oxygen concentration over time. Measured values are reported in Figure 2 and show a quick decrease in the first 3 days of the process, which decreased close to 0 μg/L after that time. No differences could be detected between white and red samples so this trend was matrix independent.

Table S5 presents the concentration of VCCs during the accelerated aging process in Gewürztraminer samples. Even though 44 analytes were quantified, only the compounds with a significant variation during the aging are shown. The hidden analytes did not identify any trend or were stably below the limit of quantification. Carbonyls which showed the most important variations (*p* < 0.05) were 2-butanone, 3-methyl-2-butanone (fruity aroma), 3-penten-2-one (fishy and phenolic aroma), 2-hexanone (toasty, caramel, and woody aroma), 2-cyclohexen-1-one (roasted and savory), 2-propenal (burnt fat), 4-methyl-4-methylthio-2-pentanone (sulphureous), propanal (fruity odor, fresh green aroma), butanal (chocolate-type odor), pentanal (nut-like odor, dry fruit), hexanal (herbaceous, cut grass, unripe fruit odor), methional (potato chips odor), 2-furfural (almond-like odor), 5-methyl-2-furfural (spicy-sweet and caramel-like odor), 2-methylpropanal (fermented, overripe, malty odor), 2-methylbutanal and 3-methylbutanal (peach-like flavor, cheesy, unripe banana odor), and phenylacetaldehyde (sweet, honey-like, rose aroma).^38,55,56^

The production of carbonyls can be due to many chemical or microbiological processes, depending on the operating conditions. During winemaking, the amount of oxygen is reduced so that the VCCs are mainly produced by microbiological processes, while during the postbottling evolution, chemical oxidation is the principal responsible for the formation of carbonyls. In these experiments, accelerated aging aims to repeat what happens in the bottles over time, so the VCCs that accumulate are only the product of chemical and non-microbiological processes.

**Figure 3:**
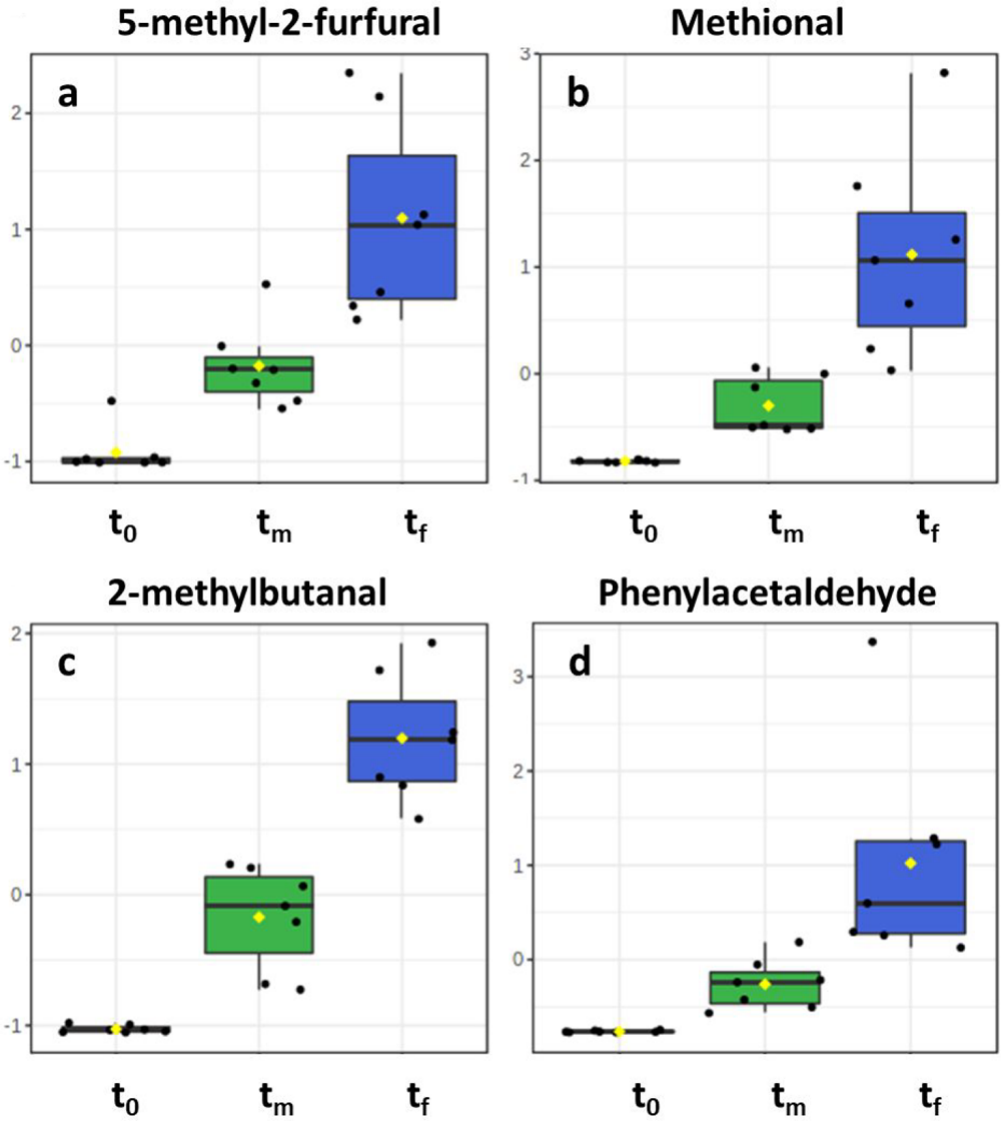
Evolution of 5-methyl-2-furfural (a), methional (b), 2-methylbutanal (c), and phenylacetaldehyde (d) in Gewürztraminer samples. Autoscaled values.

Furans are originated from the dehydration of carbohydrates (2-furfural from pentoses and 5-methyl-2-furfural from rhamnose) with consequent cyclization in Maillard-type reactions.^55^ The values of these furans measured after 2 weeks of an accelerated aging process were similar to those found by Moreira et al. in port wines aged for 10 years.^40^ Furan accumulation is usually related to browning phenomena and can be used as a parameter to measure age in oxidized wines.^55^ The trend for 5-methyl-2-furfural is shown in Figure 3a.

Other molecules whose production was significant were Strecker aldehydes, such as methional (Figure 3b), 2-methylbutanal (Figure 3c), 3-methylbutanal, and phenylacetaldehyde (Figure 3d). These compounds are mainly formed via amino acid decarboxylation and deamination^28^ or, to a lesser extent, through fusel alcohol transformation.^9,57^ Their accumulation is facilitated by a negligible amount of oxygen in wine, the condition that in this experiment took place after the first days of warming. Based on the models proposed by Bueno et al., the accumulation of non-aromatic Strecker aldehydes is directly related to their amino acid precursor concentration and inversely related to aldehyde reactive polyphenols (ARPs), which are expected to be in a negligible amount in a white wine like Gewürztraminer.^9^ The same model indicates that phenylacetaldehyde behaves in a different way, which is less related to ARPs because of its different synthetic pathway. The presented data are in strong accordance with the latest models, and the formation of Strecker aldehydes was as high as expected.

**Figure 4:**
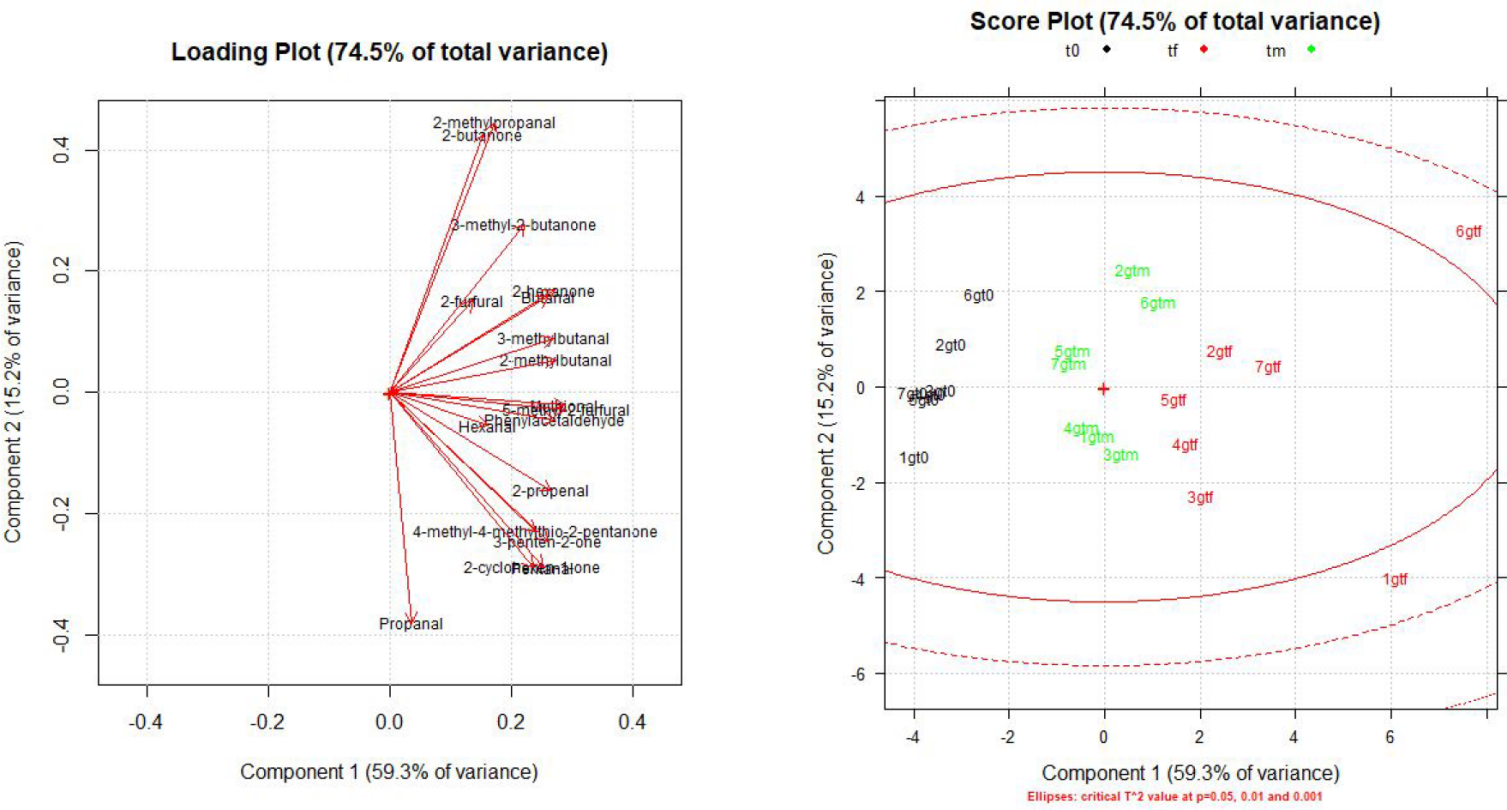
Loading plot and score plot obtained from PCA in Gewürztraminer samples.

Principal component analysis (PCA) shows loadings of 59.3% for PC1, 15.22% for PC2, and lower values for other PCs, making negligible their contribution to the explained variance. In the score plot, a sharp separation can be detected along PC1 where samples distribute in three groups that correspond to all aging steps (Figure 4). It can also be noted how samples increase their distances along the PCs, emphasizing their different aging potential.

The amount of VCCs in Teroldego samples is reported in Table S6; even in this case, only analytes with a significant variation are shown: 2-butanone, 3-methyl-2-butanone, 3-penten-2-one, 2-hexanone, E-2-butenal, 4-(methylthio)-2-butanone, 4-methyl-4-methylthio-2-pentanone, propanal, butanal, pentanal, hexanal, heptanal, methional, 2-furfural, 5-methyl-2-furfural, 2-methylpropanal, 2-methylbutanal, 3-methylbutanal, 2-methylpentanal, and benzaldehyde. In this case, E-2-butenal (burnt fat), E-2-pentenal (fruity odor), E-2-hexenal (fresh green odor), 4-(methylthio)-2-butanone (vegetative, potato, earthy, tomato odor), heptanal (fruity, oily greasy odor), and benzaldehyde (almond flavor) gave a significant variation (*p* < 0.05), whereas 2-cyclohexen-1-one, 2-propenal, and phenylacetaldehyde were not significant.

**Figure 5:**
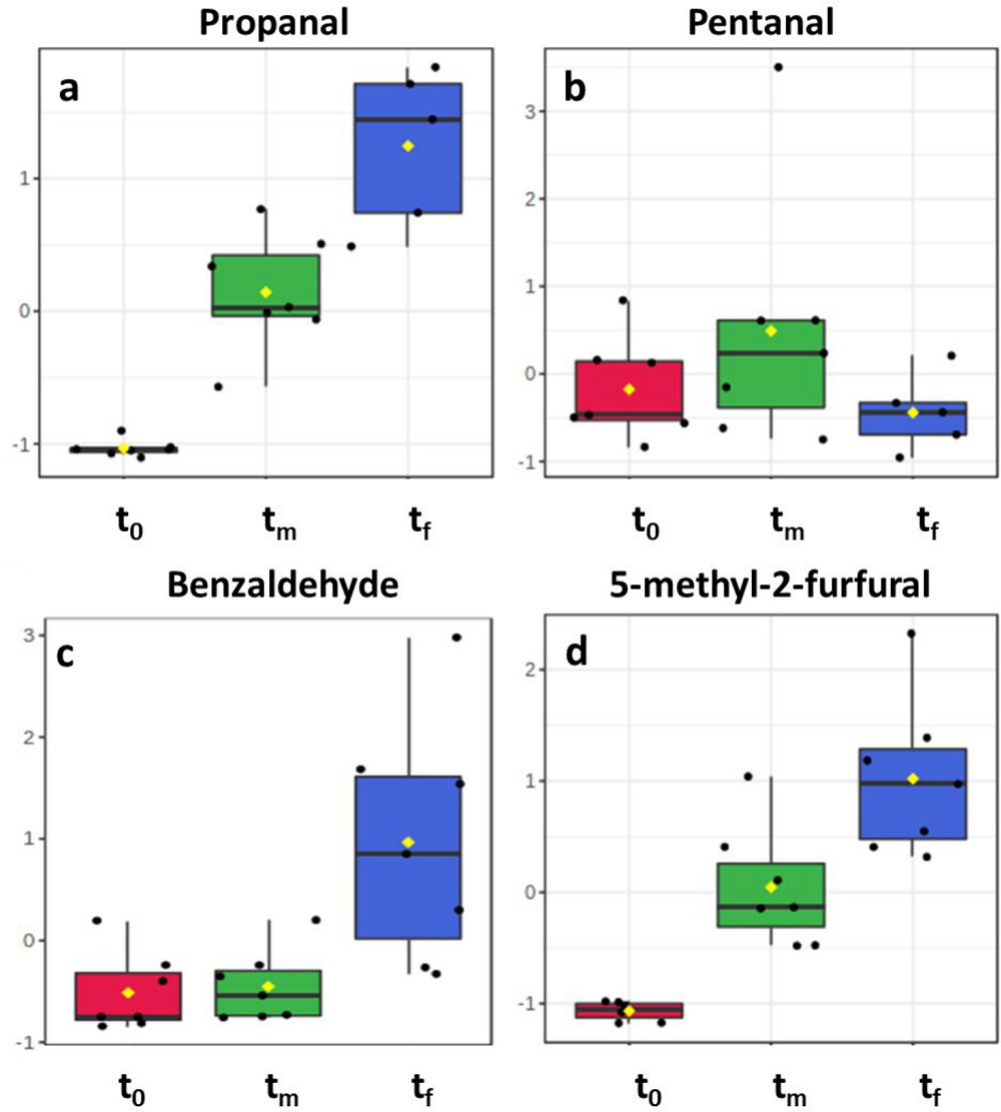
Evolution of propanal (a), pentanal (b), benzaldehyde (c), and 5-methyl-2-furfural (d) in Teroldego samples. Autoscaled values.

Teroldego showed a more important increase in the concentration of short chain linear aldehydes such as propanal (Figure 5a) and butanal compared to Gewürztraminer, whereas pentanal (Figure 5b), hexanal, and heptanal had a nonlinear behavior. These analytes accumulate during the first week and then decrease during the second week; since aldehydes are the midway between alcohol and carboxyl acid, and these are involved in esterification reactions especially, which are boosted by temperature and pressure, it is reasonable to assume that the system moved in this direction.^43,58^ Alternatively, the aldehydes accumulating in red wines could also be consumed in electrophilic addition to flavonoid reactions.

A similar trend was also observed for hexanal in 1G, 3G, and 5G Gewürztraminer samples (Table S5). Benzaldehyde (Figure 5c) behaves still in the same way as Strecker aldehydes in Gewürztraminer, probably because of the balance between the low protection due to the reduced amount of SO_2_ and the preservative effect attributed to the higher presence of ARPs; the lower concentration of SO_2_ in red wines could be a key factor in the different oxidative evolution between Teroldego and Gewürztraminer samples.^59^ This last hypothesis is also supported by the stronger increase detected in furans^28^ (Figure 5d).

**Figure 6:**
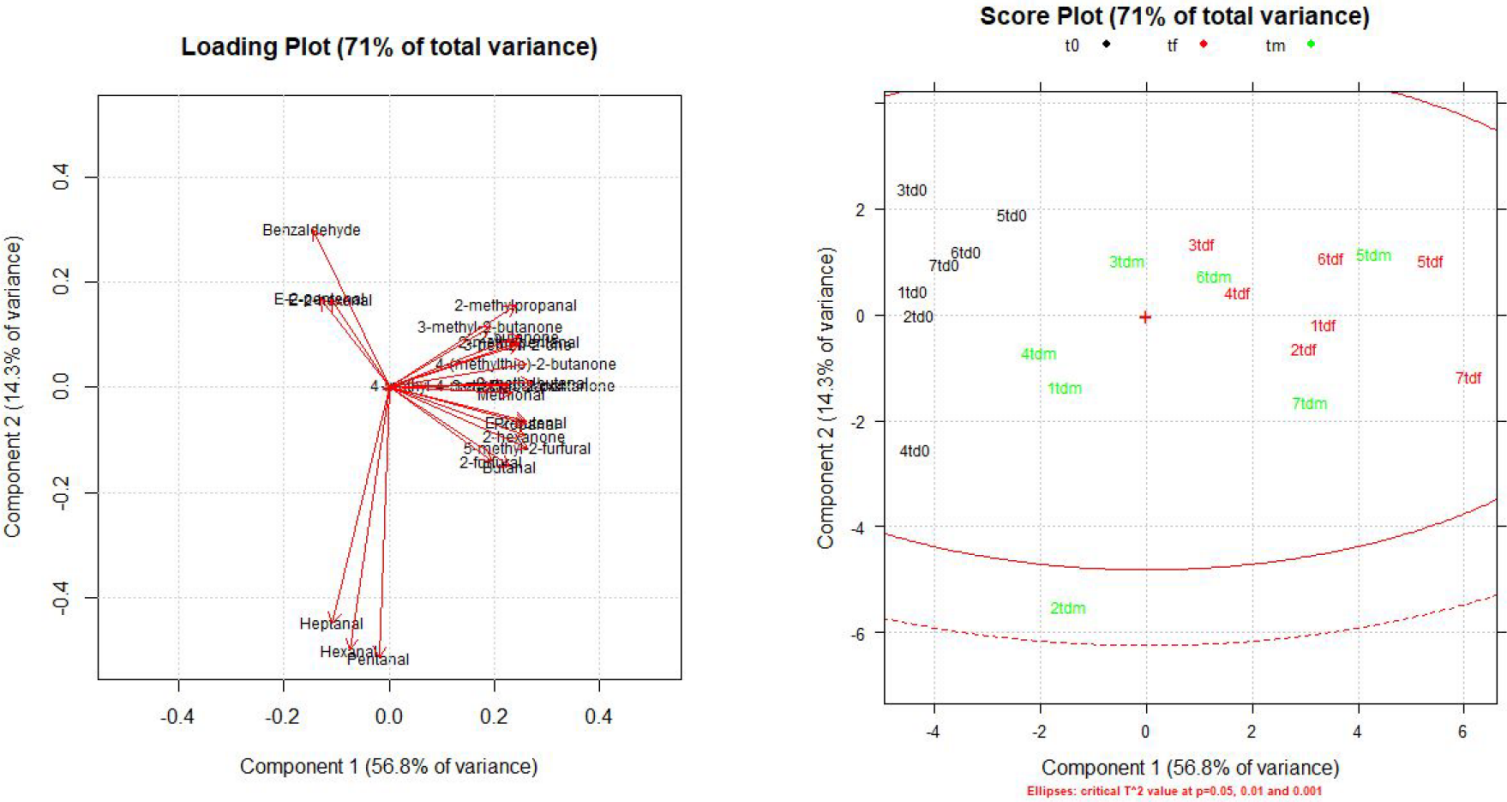
Loading plot and score plot obtained from PCA in Teroldego samples.

PCA (Figure 6) confirms the similarities between Gewürztraminer and Teroldego samples and highlights some differences. Like in white wines, PC1 (56.8%) and PC2 (14.3%) are the only relevant components, since they explain up to 71% of all variance. Samples coming from different aging steps are well separated from the others, but with a broader distribution along PC2 compared to Gewürztraminer PCA. In addition, the loading plot shows that most analytes are directly correlated with PC1, while the compounds with a nonlinear behavior mentioned before are more correlated to PC2.

Based on the results presented above, the method demonstrated high robustness, usability in a wide range of concentrations, good precision even for analytes under micrograms per liter, and a reduced matrix effect. The use of HS-SPME coupled with minimized volumes makes for a negligible environmental impact per sample. Its performance, productivity, and robustness, coupled with its green character, make of this method a versatile tool that could be used in routine analysis for monitoring the correct winemaking and the evolution of wine during aging and as a proper bottling and storing process control.

## Supporting Information Available

Figures reporting studies on derivatization at different times in G1 and G2 samples (Figures S1 and S2, respectively) with and without the addition of SO_2_ and acetaldehyde, tables on validation experiments (Tables S1–S4), and numeric results in accelerated aging samples (Tables S5 and S6) (PDF)

## Abbreviations Used

VCCs: volatile carbonyl compounds
PFBHA: O-(2,3,4,5,6-pentafluorobenzyl) hydroxylamine hydrochloride
HS-SPME: head space solid phase microextraction
