# Supplementary material for: Development of a Fully Automated Method HS-SPME-GC-MS/MS for the Determination of Odor-Active Carbonyls in Wines: a “Green” Approach to Improve Robustness and Productivity in the Oenological Analytical Chemistry
Source: J Agric Food Chem. 2023 Feb 27;72(4):1995–2007. doi: 10.1021/acs.jafc.2c07083 (PMC10835727; doi:10.1021/acs.jafc.2c07083)
Supplement: Supplementary file 1 — jf2c07083_si_001.pdf [file jf2c07083_si_001.pdf]

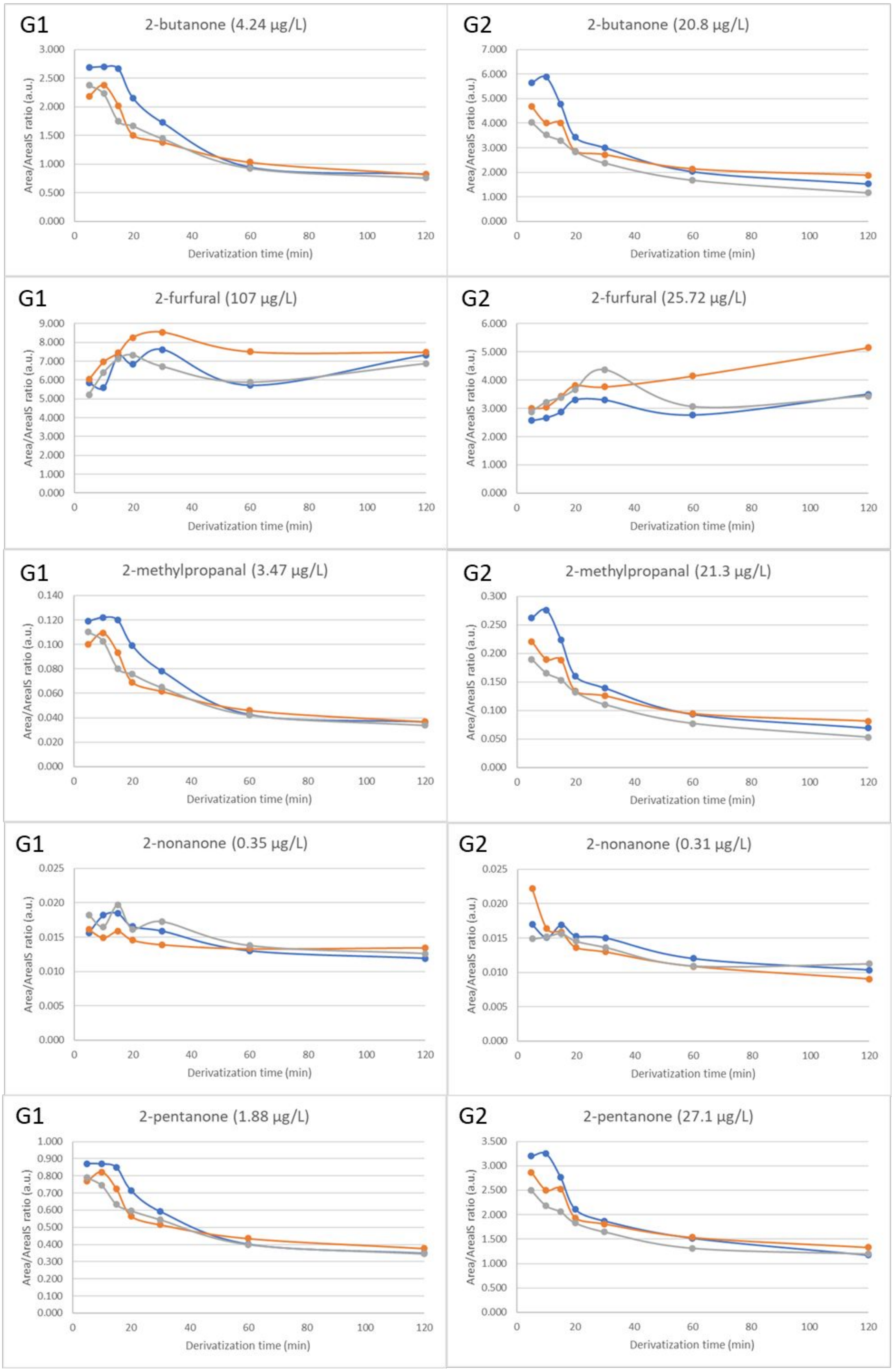

Figure S1. Compound area/internal standard area ratio for detectable analytes (1st half) in the studies of derivatization times for G1 and G2 samples. Untreated wines are reported in blue (●), wines spiked with 40 mg/L acetaldehyde are reported in orange (●), and wines spiked with 20 mg/L SO_2 are reported in grey (●). In brackets, concentration measured at 10 minutes for untreated wine.

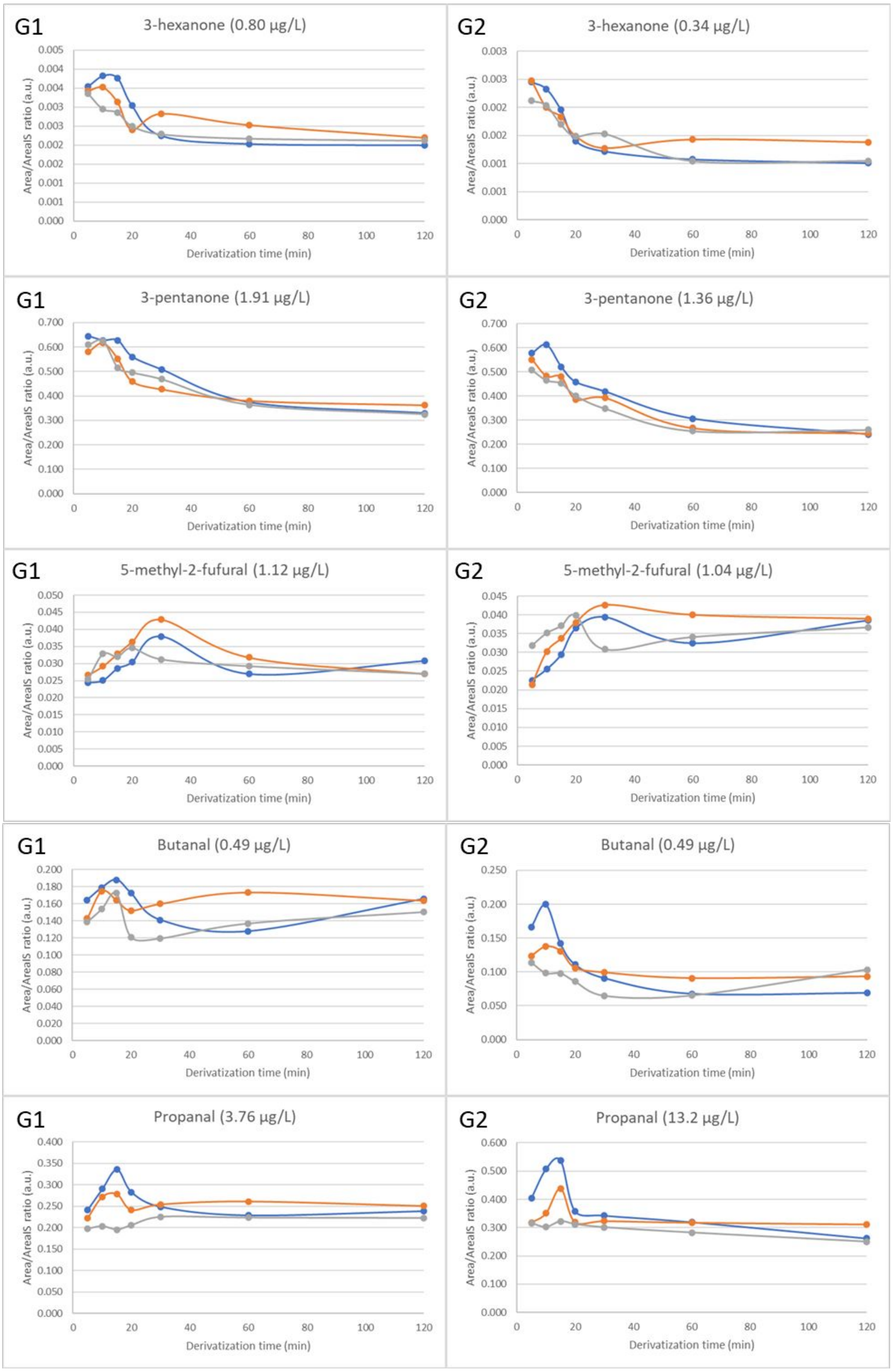

Figure S2. Compound area/internal standard area ratio for detectable analytes (2nd half) in the studies of derivatization times for G1 and G2 samples. Untreated wines are reported in blue (●), wines spiked with 40 mg/L acetaldehyde are reported in orange (●), and wines spiked with 20 mg/L SO_2 are reported in grey (●). In brackets, concentration measured at 10 minutes for untreated wine.

Analyte	*Intra-day repeatability*		*Inter-day repeatability*	
	Avg. Conc.	RSD (%)	Avg. Conc.	RSD (%)
2-butanone	0.22	13.33	0.29	7.52
2-cyclohexen-1-one	0.13	17.06	0.16	8.41
2-decanone	0.19	5.26	0.20	8.02
2-furfural	0.65	8.95	0.59	24.22
2-heptanone	0.17	25.33	0.20	14.94
2-hexanone	0.15	23.83	0.23	20.97
2-methyl-3-pentanone	0.18	5.61	0.21	20.81
2-methylbutanal	0.36	25.01	0.44	9.26
2-methylpentanal	0.21	11.02	0.28	25.21
2-methylpropanal	0.22	13.02	0.33	13.14
2-nonanone	0.18	5.35	0.23	20.53
2-octanone	0.16	9.61	0.21	15.50
2-pentanone	0.22	13.08	0.31	12.70
2-propenal	0.87	22.95	1.02	17.56
2-undecanone	0.19	7.77	0.21	10.11
3-hexanone	0.25	7.55	0.32	13.43
3-methyl-2-butanone	0.20	11.95	0.29	16.53
3-methyl-2-butenal	0.16	14.87	0.22	13.92
3-methylbutanal	0.44	26.37	0.53	16.61
3-methylthio-2-butanone	0.16	12.21	0.21	13.67
3-pentanone	0.20	9.93	0.27	15.71
4-(methylthio)-2-butanone	0.29	24.40	0.42	18.55
4-heptanone	0.15	8.01	0.17	11.81
4-methyl-2-pentanone	0.17	12.17	0.23	18.12
4-methyl-4-methylthio-2-pentanone	0.20	8.43	0.32	18.90
5-methyl-2-furfural	0.19	8.65	0.22	26.61
6-methyl-5-hepten-2-one	0.18	8.96	0.24	17.16
Benzaldehyde	0.18	21.02	0.28	13.64
Butanal	0.56	9.86	0.70	7.91
E-2-butenal	0.15	10.53	0.28	19.94
E-2-decenal	0.20	15.93	0.17	18.24
E-2-heptenal	0.17	25.58	0.19	12.94
E-2-hexenal	0.23	19.43	0.23	25.11
E-2-nonenal	0.23	14.65	0.22	18.92
E-2-octenal	0.17	4.28	0.24	10.61
E-2-pentenal	0.14	14.42	0.22	11.01
Heptanal	0.23	7.30	0.27	28.5
Hexanal	0.30	21.77	0.29	26.4
Methional	0.16	15.71	0.26	27.03
Nonanal	0.15	22.36	0.23	7.07
Octanal	0.41	24.14	0.79	25.4
Pentanal	0.23	15.74	0.35	15.00
Phenylacetaldehyde	0.27	19.96	1.24	23.7
Propanal	0.16	15.21	0.32	10.30

Table S1. Intra-day and inter-day repeatability for laboratory sample prepared at 0.2 µg/L.

Analyte / Concentration level	*Intra-day repeatability RSD (%)*			*Inter-day repeatability RSD (%)*		
	0.2 µg/L	***5 µg/L***	***50 µg/L***	***0.2 µg/L***	***5 µg/L***	***50 µg/L***
2-butanone	10.47	5.91	11.48	11.66	5.77	6.49
2-cyclohexen-1-one	18.34	12.49	16.54	17.77	16.78	12.43
2-decanone	13.43	17.22	11.04	19.39	24.51	12.35
2-furfural	5.78	24.26	11.33	10.73	9.62	11.17
2-heptanone	26.86	6.21	18.96	27.46	8.89	22.17
2-hexanone	13.34	10.52	21.64	9.19	7.31	13.84
2-methyl-3-pentanone	3.96	7.96	11.47	6.27	11.92	11.25
2-methylbutanal	8.64	15.75	7.82	7.72	28.56	7.18
2-methylpentanal	11.03	10.22	11.56	2.62	5.99	11.64
2-methylpropanal	9.87	9.75	10.74	4.45	5.62	10.68
2-nonanone	3.71	7.34	17.83	4.86	10.75	8.93
2-octanone	8.56	2.82	17.08	11.14	7.04	22.53
2-pentanone	4.67	18.22	12.22	5.44	11.45	12.44
2-propenal	12.4	4.67	5.54	23.31	19.01	2.79
2-undecanone	13.52	24.8	22.06	22.56	26.00	19.43
3-hexanone	1.82	7.35	5.05	2.15	5.57	5.01
3-methyl-2-butanone	7.47	19.90	11.54	6.96	28.31	11.66
3-methyl-2-butenal	10.82	19.04	3.75	14.24	10.84	5.63
3-methylbutanal	18.06	5.94	8.61	6.27	22.74	8.53
3-methylthio-2-butanone	13.93	11.35	17.24	22.41	9.23	23.56
3-pentanone	5.81	20.22	11.1	5.05	27.26	11.37
4-(methylthio)-2-butanone	13.87	7.97	20.33	2.93	4.68	21.81
4-heptanone	14.02	15.41	25.2	9.11	21.18	25.39
4-methyl-2-pentanone	12.3	10.60	17.84	7.24	15.82	21.87
4-methyl-4-methylthio-2-pentanone	8.27	8.21	3.87	7.66	8.24	4.37
5-methyl-2-furfural	14.37	14.7	10.65	10.75	13.93	10.31
6-methyl-5-hepten-2-one	7.26	8.12	20.66	1.47	20.32	15.49
Benzaldehyde	12.5	5.35	18.14	14.62	18.41	6.23
Butanal	20.95	7.01	9.64	1.51	7.96	10.06
E-2-butenal	26.36	27.77	13.09	23.16	24.78	13.9
E-2-decenal	19.23	12.13	52.07	7.69	111.0	2.69
E-2-heptenal	20.38	17.41	13.24	5.46	13.61	13.97
E-2-hexenal	14.94	12.84	11.26	3.51	20.44	11.79
E-2-nonenal	16.77	27.82	19.01	14.61	29.85	18.61
E-2-octenal	10.27	27.82	18.73	19.92	28.71	23.49
E-2-pentenal	17.12	9.30	7.74	27.14	15.37	13.24
Heptanal	15.50	28.40	8.15	27.98	28.95	13.12
Hexanal	25.52	24.91	18.02	16.79	13.53	15.45
Methional	10.65	11.88	14.21	14.07	6.86	14.19
Nonanal	21.88	14.76	10.55	15.35	13.43	10.87
Octanal	28.77	14.05	11.14	15.52	26.32	14.18
Pentanal	5.16	19.76	21.06	9.65	21.22	18.42
Phenylacetaldehyde	27.04	11.91	2.98	29.91	14.61	7.61
Propanal	19.79	16.32	6.05	17.86	27.54	6.69

Table S2. Intra-day and inter-day repeatability (RSD%) at 0.2, 5 and 50 µg/L in red wine matrix.

Analyte	***Sfursat***		***Sfursat + spike 5 µg/L***			***Sfursat + spike 50 µg/L***		
	Avg. Conc.	RSD (%)	Avg. Conc.	RSD (%)	Recovery %	Avg. Conc.	RSD (%)	Recovery %
2-butanone	1.02	20.30	6.29	7.13	92.62	44.22	0.72	87.98
2-cyclohexen-1-one	< 0.05	n.a.	3.79	8.62	76.04	41.81	5.85	83.61
2-decanone	< 0.05	n.a.	3.22	5.93	85.75	38.41	10.91	78.94
2-furfural	49.2	4.56	54.19	4.89	99.62	119.2	3.01	116.8
2-heptanone	< 0.05	n.a.	5.07	4.22	107.1	62.92	4.91	122.9
2-hexanone	< 0.05	26.36	5.47	4.76	108.8	62.42	5.16	120.6
2-methyl-3-pentanone	0.14	31.05	5.52	5.35	107.5	60.67	4.49	121.0
2-methylbutanal	13.6	7.27	19.71	3.49	110.5	71.9	3.14	116.8
2-methylpentanal	0.05	5.68	9.97	3.46	99.25	107.0	5.22	118.9
2-methylpropanal	4.46	9.98	13.15	5.89	86.89	47.46	0.94	75.99
2-nonanone	0.09	5.83	5.08	16.9	99.62	51.21	10.51	102.2
2-octanone	< 0.05	n.a.	4.10	5.93	82.13	53.63	5.25	107.2
2-pentanone	< 0.05	n.a.	6.42	4.72	117.4	59.03	2.94	119.3
2-propenal	< 0.05	n.a.	1.56	7.61	115.8	40.57	4.77	82.71
2-undecanone	0.06	25.13	4.13	8.74	82.09	42.24	34.52	74.38
3-hexanone	3.03	1.11	8.91	0.67	117.5	72.66	3.92	116.1
3-methyl-2-butanone	< 0.05	n.a.	5.19	5.11	125.1	66.35	3.61	112.3
3-methyl-2-butenal	0.08	n.a.	6.26	2.92	123.5	87.05	6.11	124.2
3-methylbutanal	87.4	4.29	92.9	4.82	108.7	136.1	3.57	123.9
3-methylthio-2-butanone	< 0.05	n.a.	4.09	10.31	81.76	54.27	5.39	108.5
3-pentanone	2.68	8.72	8.50	18.33	116.4	64.65	4.02	123.9
3-penten-2-one	1.82	4.02	5.64	2.74	85.32	51.72	4.97	99.83
4-(methylthio)-2-butanone	0.43	6.63	5.12	10.61	93.71	60.35	2.91	119.8
4-methyl-2-pentanone	0.11	4.93	4.54	3.66	88.61	56.11	5.11	111.7
4-methyl-4-methylthio-2-pentanone	0.21	5.17	5.71	4.76	109.7	60.99	3.84	117.9
5-methyl-2-furfural	0.22	14.9	4.07	12.62	76.92	48.07	4.71	95.73
6-methyl-5-hepten-2-one	2.12	3.98	6.57	20.86	89.08	68.59	12.41	120.8
Benzaldehyde	< 0.05	n.a.	3.12	28.62	90.72	66.23	4.54	122.9
Butanal	23.0	0.87	28.94	2.34	116.1	78.71	1.89	111.2
E-2-butenal	0.22	29.65	6.10	14.41	117.6	50.38	15.09	100.3
E-2-decenal	215	11.26	221.1	11.37	119.8	262.1	3.71	84.01
E-2-heptenal	< 0.05	n.a.	4.40	72.17	85.27	40.58	49.71	83.88
E-2-hexenal	0.15	22.51	6.46	4.73	120.6	53.8	3.27	103.7
E-2-nonenal	0.43	31.69	5.75	46.02	106.4	43.38	26.74	81.92
E-2-octenal	0.15	33.85	6.36	47.11	124.2	51.29	6.32	102.2
E-2-pentenal	< 0.05	n.a.	4.80	37.22	97.55	63.69	2.21	127.5
Heptanal	0.14	10.94	2.94	6.71	93.26	30.04	12.02	99.66
Hexanal	0.11	26.09	4.56	3.15	89.08	44.37	6.85	88.52
Methional	< 0.05	n.a.	4.79	47.24	95.93	63.73	4.81	119.6
Nonanal	< 0.05	n.a.	5.71	45.09	115.5	64.46	6.85	117.3
Octanal	2.11	10.8	6.32	10.11	84.16	60.3	11.91	122.8
Pentanal	1.35	10.24	6.15	15.51	95.96	57.88	2.78	113.2
Phenylacetaldehyde	2.26	5.87	8.37	8.88	122.2	61.51	20.12	118.5
Propanal	< 0.05	n.a.	5.97	5.81	123.7	63.39	4.04	127.2

Table S3. Intra-day repeatability and recovery evaluation of the Sfursat sample spiked at 5 and 50 µg/L.

**Analyte\Sample name**	**G00**	**G05**	**G50**	**G75**	**T00**	**T05**	**T50**	**T75**
2-butanone	0.63	52.4	138	150	100	107	178	207
2-cyclohexen-1-one	0.31	0.352	0.422	0.475	0.26	0.263	0.303	0.331
2-furfural	2266	2224	3948	3473	887	1121	1141	3185
2-heptanone	< 0.05	0.21	1.65	1.07	0.28	0.22	< 0.05	1.01
2-hexanone	< 0.05	0.35	0.73	0.96	0.39	0.54	0.27	4.45
2-methylbutanal	35.0	60.7	448	499	209	180	268	318
2-methylpentanal	0.27	0.45	0.93	2.06	1.57	1.34	1.65	3.79
2-methylpropanal	< 0.05	44.2	121	129	86.1	79.4	68.1	187
2-octanone	< 0.05	< 0.05	0.35	0.27	< 0.05	< 0.05	< 0.05	0.23
2-pentanone	1.51	9.76	16.2	17.2	23.2	20.8	4.11	15.9
2-propenal	79.9	244.4	942	1033*	725	801	828	810
3-hexanone	2.65	2.01	3.39	3.29	2.33	1.93	2.20	3.76
3-methyl-2-butanone	< 0.05	0.42	29.8	30.7	14.4	16.5	98.1	79.0
3-methyl-2-butenal	< 0.05	0.11	8.83	8.14	0.52	0.67	1.74	4.64
3-methylbutanal	40.0	73.5	6179*	6971*	348	296	1184*	1534*
3-pentanone	0.38	7.63	8.17	8.21	40.5	39.7	38.9	37.0
4-(methylthio)-2-butanone	0.29	0.40	1.52	1.26	0.36	0.32	0.21	0.78
4-methyl-2-pentanone	0.32	0.44	1.30	1.29	0.69	0.63	0.70	1.28
4-methyl-4-methylthio-2-pentanone	0.52	1.00	1.38	1.50	0.65	0.48	1,29	1.43
5-methyl-2-furfural	24.2	29.1	30.0	27.4	8.72	12.2	19.9	29.5
Benzaldehyde	1.0	1.82	74.0	77.6	5.29	5.72	55.4	97.6
Butanal	5.76	15.2	138	152	23.1	17.9	14.4	58.7
E-2-butenal	0.30	0.46	449	437	19.6	17.6	72.2	95.5
E-2-hexenal	< 0.05	< 0.05	1.39	1.18	< 0.05	< 0.05	< 0.05	0.07
Heptanal	< 0.05	< 0.05	2.75	2.43	< 0.05	0.45	0.99	1.37
Hexanal	0.89	5.43	66.4	51.3	1.28	2.4	10.5	18.5
Methional	2.12	5.21	483*	472	0.56	1.06	0.79	< 0.05
Pentanal	1.29	0.87	38.9	44.4	9.05	7.14	4.99	24.9
Phenylacetaldehyde	< 0.05	3.36	5.77	19.8	7.59	8.45	10.5	12.5
Propanal	76.9	244	954	1045*	624	632	842	836

Table S4. Concentration of accumulating VCCs in Gewürztraminer and Teroldego samples during accelerated aging for 5 weeks at 50°C with different head-space volumes, in µg/L; values with * were semi-quantified over the maximum point of the calibration curve.

Analyte/ Sample name	2-butanone	3-methyl-2-butanone	3-penten-2-one	2-hexanone	2-cyclohexen-1-one	2-propenal	4-methyl-4-methylthio-2-pentanone	Propanal	Butanal
O.T.[49–52]	440	< 0.05	1.5	250	< 0.05	17	< 0.05	9.5	9.0
$1gt_0$	< 0.05	0.47	0.33	< 0.05	< 0.05	0.39	1.67	27.0	18.4
$1gt_m$	13.1	1.16	7.62	0.14	0.37	7.69	32.5	15.4	91.8
$1gt_f$	17.9	2.08	36.0	0.27	0.96	19.0	120	16.4	104
$2gt_0$	30.08	2.28	1.06	< 0.05	< 0.05	0.62	0.74	2.11	29.1
$2gt_m$	56.5	3.81	7.02	0.27	0.19	4.02	30.0	1.60	88.8
$2gt_f$	67.9	4.26	9.94	0.22	0.35	9.75	37.6	0.44	98.2
$3gt_0$	3.91	0.78	0.64	0.06	0.10	1.25	0.89	< 0.05	36.4
$3gt_m$	9.92	0.94	9.01	0.30	0.60	14.2	18.0	8.73	46.0
$3gt_f$	18.3	2.02	12.5	0.16	0.28	16.6	61.4	31.7	55.9
$4gt_0$	6.74	1.23	0.08	< 0.05	< 0.05	0.46	7.39	4.26	7.62
$4gt_m$	18.6	2.40	7.23	0.22	0.25	2.50	75.2	15.7	25.2
$4gt_f$	23.0	2.87	10.7	0.23	0.49	6.08	95.1	12.1	40.3
$5gt_0$	15.10	0.86	0.36	< 0.05	0.056	0.77	1.00	8.85	12.06
$5gt_m$	33.7	2.12	5.37	0.21	0.26	2.72	40.5	2.12	39.6
$5gt_f$	42.9	3.22	10.3	0.25	0.43	4.33	56.6	21.1	48.5
$6gt_0$	68.1	1.78	1.24	0.06	0.07	0.50	< 0.05	3.85	47
$6gt_m$	81.6	2.01	5.15	0.28	0.21	6.13	35.3	11.5	92.3
$6gt_f$	120	4.52	15.3	0.58	0.38	17.5	66.2	0.15	134
$7gt_0$	4.26	0.49	0.67	< 0.05	< 0.05	0.27	1.69	< 0.05	21.4
$7gt_m$	18.9	1.86	9.34	0.25	0.15	1.03	19.0	7.60	54.6
$7gt_f$	33.1	3.19	15.8	0.34	0.32	12.5	33.2	3.97	103

Pentanal	Hexanal	Methional	2-furfural	5-methyl-2-furfural	2-methylpropanal	2-methylbutanal	3-methylbutanal	Phenylacetaldehyde
12	97	0.2	3000	6000	0.1	1.5	0.15	4.0
0.13	0.50	0.70	118	26.4	1.00	1.61	15.6	9.15
20.8	46.4	160	816	59.9	18.0	83.5	959	973
90.1	37.5	466	4861	431	23.8	419	2485*	2918*
< 0.05	0.69	2.81	142	4.26	35.2	15.0	96.2	33.3
4.48	8.74	57.6	1431	104	66.8	280	603	1432*
22.4	34.6	268	5430*	274	67.6	509	1335*	2522*
0.19	25.3	4.74	266	6.02	6.74	6.04	97.2	21.7
15.6	92.3	127	786	68.5	12.8	74.0	936	893
44.8	22.6	341	3392	158	23.5	363	2861*	2431*
0.44	< 0.05	< 0.05	205	68.1	8.18	3.97	22.0	13.6
12.1	4.28	56.2	2187	129	22.8	286	541	1896*
29.8	11.7	156	3098	173	27.5	496	1504*	3205*
0.58	0.13	2.91	166	< 0.05	17.7	6.89	52.5	20.6
7.55	14.5	63.08	991	88.1	40.6	188	520	1218*
19.5	8.99	192	3813	188	50.9	433	1549*	2512*
< 0.05	0.85	5.48	351	1.03	78.2	17.7	124	33.8
11.6	46.6	150	3896	197	96.7	216	1352*	1469*
43.4	51.9	658	7417*	404	130	660	6579*	3147*
0.11	0.25	0.46	138	24.6	5.97	2.33	31.2	8.38
5.80	3.68	59.2	1193	103	23.9	249	533	1514*
23.2	18.7	376	5568*	263	41.3	614	2505*	4053*

Table S5. Concentration in µg/L of significant VCCs in Gewürztraminer samples before the accelerated aging (t_0), after 2.5 weeks (t_m), and after 5 weeks (t_f); values with * were semi-quantified over the maximum point of the calibration curve. Odor thresholds indicated were reported from the ones in literature which were measured in wine (preferred), beer, or water.

Analyte/ Sample name	2-butanone	3-methyl-2-butanone	3-penten-2-one	2-hexanone	E-2-butenal	4-(methylthio)-2-butanone	4-methyl-4-methylthio-2-pentanone	Propanal	Butanal	Pentanal
O.T.[49–52]	440	< 0.05	1.5	250	0.8	< 0.05	< 0.05	9.5	9.0	12
1td0	0.14	0.77	17.8	0.14	1081	0.08	2.18	26.0	21.0	11.3
1tdm	12.7	4.35	72.5	0.34	2977	0.27	20.7	1580*	105	26.8
1tdf	22.8	8.81	198	0.46	7.33	3.01	22.8	4032*	204	21.5
2td0	7.23	0.77	39.2	<0.05	1.29	<0.05	2.90	103	115	20.9
2tdm	19.4	2.36	115	0.19	5.03	0.25	26.0	1637*	173	65.1
2tdf	33.7	4.36	202	0.28	5.27	1.97	48.6	4210*	294	14.4
3td0	11.1	0.85	20.0	<0.05	0.88	0.04	12.6	114	71.6	7.71
3tdm	21.7	3.57	116	0.28	2.34	1.53	56.3	1507*	107	10.6
3tdf	27.3	4.60	176	0.31	2.89	1.62	65.1	2289*	144	6.12
4td0	6.64	0.27	41.5	0.14	1.41	0.26	3.16	68.9	84.6	29.9
4tdm	10.9	2.13	105	0.23	2511	0.22	40.6	784	124	21.9
4tdf	23.3	3.43	293	0.41	5.82	2.80	38.0	2649*	141	13.0
5td0	26.0	5.09	83.4	<0.05	3.89	0.99	1.23	314	122	20.4
5tdm	46.6	12.8	317	0.51	7.27	3.04	37.0	2315*	179	16.7
5tdf	44.5	10.8	382	0.53	7.30	3.60	43.5	2770*	268	12.5
6td0	13.2	0.91	44.6	<0.05	2.28	0.64	2.17	111	79.1	12.2
6tdm	24.5	2.63	234	0.29	4.06	1.61	30.4	2075*	169	8.85
6tdf	35.25	3.77	388.2	0.38	6.61	2.71	35.5	3652*	110	9.58
7td0	11.9	0.45	43.6	<0.05	2.44	0.76	1.54	136	110	12.5
7tdm	24.9	2.26	140	0.51	4.95	2.87	33.7	2688*	282	26.9
7tdf	32.9	3.00	215	0.82	8.19	5.62	38.3	3940*	335	26.1

Hexanal	Heptanal	Methional	2-furfural	5-methyl-2-furfural	2-methylpropanal	2-methylbutanal	3-methylbutanal	2-methylpentanal	Benzaldehyde
97	3.0	0.2	3000	6000	0.1	1.5	0.15	< 0.05	350
37.2	5.72	8.52	766	5.74	11947	6.55	122	0.06	6.8
66.8	12.9	110	7485*	61.9	17.4	249	1371*	0.28	10.7
47.3	3.76	196	5207*	179	29.4	1092*	4017*	1.30	39.7
92.7	5.36	55.2	893	13.5	5.91	52.8	194	0.05	10.6
207	26.1	80.1	11455*	109	18.5	276	1557*	0.13	23.1
64.8	2.99	109	18427*	213	30.0	1032*	2872*	0.55	73.5
24.6	0.62	23.2	642	5.96	10.1	82.0	178	0.06	67.3
21.9	1.91	39.4	7635*	89.8	23.7	593	1560*	0.55	67.6
11.0	0.99	51.5	9760*	134	30.3	918	1659*	0.64	234
12.3	26.3	25.7	863	18.7	6.86	28.7	117	0.13	10.6
45.9	9.85	72.8	5653*	62.2	12.7	108	835	0.31	11.6
34.3	4.10	120	3898	145	30.7	730	2491*	0.83	35.9
57.2	10.3	195.6	1711	21.0	33.7	148	283	0.52	41.1
26.8	3.82	208	8846*	134	58.3	871	4074*	1.07	41.0
21.3	2.96	307	11003*	196	55.9	1057*	3802*	1.01	148
53.9	7.92	46.5	529	15.5	18.6	59.8	246	0.80	5.0
23.2	1.05	155	11827*	89.0	32.0	525	3255*	0.95	10.1
16.3	1.27	163	14459*	126	43.9	950	3336*	1.26	107
66.1	6.72	29.5	790	21.5	16.4	93.9	141	0.20	31.6
90.7	7.12	275	1473*	185	21883	941	5804*	1.13	34.4
73.4	6.63	285	5570*	289	43.0	1436*	6193*	1.58	157

Table S6. Concentration in µg/L of significant VCCs in Teroldego samples before the accelerated aging (t_0), after 2.5 weeks (t_m), and after 5 weeks (t_f); values with * were semi-quantified over the maximum point of the calibration curve. Odor thresholds indicated were measured in wine (preferred), beer, or water.
